# Artificial intelligence–enhanced mapping of the international classification of functioning, disability and health via a mobile app: a randomized controlled trial

**DOI:** 10.3389/fpubh.2025.1590401

**Published:** 2025-08-05

**Authors:** Zhandos Kurban, Didar Khassenov, Zhandos Burkitbaev, Sholpan Bulekbayeva, Azat Chinaliyev, Serik Bakhtiyar, Samat Saparbayev, Tokan Sultanaliyev, Ulzhalgas Zhunissova, Natalia Slivkina, Elena Titskaya, Luis Arias, Dana Aldakuatova, Gulfairus Yessenbayeva, Zhanerke Ermakhan

**Affiliations:** ^1^Department of Rehabilitation and Sports Medicine, NCJSC Astana Medical University, Astana, Kazakhstan; ^2^Department of Interventional Radiology, National Research Oncology Center LLP, Astana, Kazakhstan; ^3^Department of Public Health and Hygiene, NCJSC Astana Medical University, Astana, Kazakhstan; ^4^National Research Oncology Center LLP, Astana, Kazakhstan; ^5^National Scientific Center for the Development of the Social Protection Sector, Almaty, Kazakhstan; ^6^Al-Jami LLC, Astana, Kazakhstan; ^7^Department of Biostatistics, Bioinformatics and Information Technologies, NCJSC Astana Medical University, Astana, Kazakhstan; ^8^Laboratory of Medical Technology Planning and Development, Tomsk Research Institute of Balneology and Physiotherapy of the Siberian Federal Research and Clinical Center of the Federal Medical and Biological Agency, Tomsk, Russia; ^9^Department of Scientific Institute of Higher Education, Santa Cruz De La Sierra, Mexico

**Keywords:** rehabilitation, surveys and questionnaires, artificial intelligence, international classification of functioning, disability and health, mobile applications

## Abstract

**Background:**

Mobile health applications and artificial intelligence (AI) are increasingly utilized to streamline clinical workflows and support functional assessment. The International Classification of Functioning, Disability and Health (ICF) provides a standardized framework for evaluating patient functioning, yet AI-driven ICF mapping tools remain underexplored in routine clinical settings.

**Objective:**

This study aimed to evaluate the efficiency and accuracy of the MedQuest mobile application—featuring integrated AI-based ICF mapping—compared to traditional paper-based assessment in hospitalized patients.

**Methods:**

A parallel-group randomized controlled trial was conducted in two medical centers in Astana, Kazakhstan. A total of 185 adult inpatients (≥18 years) were randomized to either a control group using paper questionnaires or an experimental group using the MedQuest app. Both groups completed identical standardized assessments (SF-12, IPAQ, VAS, Barthel Index, MRC scale). The co-primary outcomes were (1) total questionnaire completion time and (2) agreement between AI-generated and clinician-generated ICF mappings, assessed using quadratic weighted kappa. Secondary outcomes included AI sensitivity/specificity, confusion matrix analysis, and physician usability ratings via the System Usability Scale (SUS).

**Results:**

The experimental group completed questionnaires significantly faster than the control group (median 18 vs. 28 min, *p* < 0.001). Agreement between AI- and clinician-generated ICF mappings was substantial (*κ* = 0.842), with 80.6% of qualifiers matching exactly. The AI demonstrated high sensitivity and specificity for common functional domains (e.g., codes 1–2), though performance decreased for rare qualifiers. The micro-averaged sensitivity and specificity were 0.806 and 0.952, respectively. Mean SUS score among physicians was 86.8, indicating excellent usability and acceptability.

**Conclusion:**

The MedQuest mobile application significantly improved workflow efficiency and demonstrated strong concordance between AI- and clinician-assigned ICF mappings. These findings support the feasibility of integrating AI-assisted tools into routine clinical documentation. A hybrid model, combining AI automation with clinician oversight, may enhance accuracy and reduce documentation burden in time-constrained healthcare environments.

**Trial registration:**

ClinicalTrials.gov, identifier NCT07021781.

## Introduction

The advancement of digital health technologies, particularly mobile applications, has profoundly transformed clinical workflows, enabling seamless integration into routine medical practice (1). Smartphones and tablets equipped with specialized software have revolutionized access to medical information, communication between healthcare providers and patients, and the overall approach to healthcare delivery. This technological shift has led to improved clinical efficiency and optimized physician working time (2, 3).

Modern mobile health applications encompass a broad spectrum of functions, including medical reference tools, drug databases, health monitoring, telemedicine services, and remote patient monitoring (4). These applications consistently demonstrate effectiveness in enhancing patient care quality and reducing appointment durations (5). Furthermore, smartphones have become widespread across all demographics, including older adults, which facilitates the integration of mobile apps into rehabilitation (6) and opens avenues for remote management of care—a particularly vital benefit in rural regions with workforce shortages (7).

Clinicians today face a critical challenge: severely limited patient appointment times, a problem worsened by staff shortages and increasing demands on healthcare systems (8). This challenge is particularly acute in Kazakhstan, where general practitioners have only 15 min per patient, while specialists receive 20 min (9). Within this brief window, physicians must conduct interviews, perform examinations, and complete all necessary medical documentation. These time constraints significantly impede thorough patient assessment and increase the risk of medical errors due to insufficient time for comprehensive clinical decision-making.

Artificial intelligence (AI) technologies offer promising solutions in this context (10, 11). AI has substantial potential to accelerate clinical assessments, support clinical decision-making, and improve assessment accuracy, particularly in functional evaluation and mapping using frameworks like the ICF (12). However, the effectiveness of AI-driven tools in functional health classification remains largely unexplored, especially in real-world clinical settings. Research in this area is essential to determine AI’s effectiveness in ICF mapping and the practical value of its recommendations for clinicians.

The ICF is a foundational tool in modern medicine, offering a holistic perspective on human health that transcends the traditional disease-centric medical model, which primarily focuses on diagnoses and pathophysiological disorders (13). It provides a universal, standardized language for specialists globally, fostering effective interdisciplinary communication (12). ICF mapping involves the systematic assignment of specific alphanumeric codes and qualifiers from the ICF classification system to categorize various domains of human functioning. This comprehensive mapping process encompasses three key areas: “Body Functions and Structures” (such as physiological functions and anatomical parts), “Activities and Participation” (including task execution and involvement in life situations), and “Environmental Factors” (encompassing physical, social, and attitudinal surroundings). The primary goal of ICF mapping is to provide a holistic understanding of an individual’s functional status that complements traditional disease diagnoses (14). In contrast to the disease-oriented diagnostic classification of ICD, the ICF focuses on an individual’s functional capacity, which is paramount for planning and evaluating the efficacy of rehabilitation interventions (15). ICF has value across many clinical areas. In geriatrics, it helps in the comprehensive assessment of older adults (16). In neurology, the ICF framework is used for rehabilitation after stroke, brain injury and treatment of neurodegenerative diseases (17). It is also used in orthopedics and traumatology to assess functional limitations after injury (18). In addition, fields such as psychiatry, pediatrics, and social medicine use ICF for holistic patient assessment (19–21). Notably, health care and insurance systems use ICF mapping as a basis for making decisions about insurance coverage and resource allocation (22).

Due to significant time constraints in clinical practice, the challenges of manual ICF mapping, and the untapped potential of AI in functional health classification, a more integrated solution was needed (8, 10). To address this critical gap, we developed MedQuest, a free mobile application for our clinic (23). This program is designed to streamline the administration of large volumes of functional status questionnaires while maintaining accurate ICF mapping. Automated ICF mapping speeds up the diagnostic process and increases the objectivity of assessments (24), while the mobile questionnaire format minimizes transcription errors and increases patient accessibility (25). MedQuest also optimizes data handling by providing built-in analytical tools and secure cloud storage in compliance with international and local data protection standards (26–28). Furthermore, the transition to electronic questionnaires also contributes to reduced environmental impact by decreasing paper and other resource consumption (29). Crucially, MedQuest prioritizes data security, incorporating features like encryption and authentication to ensure medical information confidentiality in compliance with major regulatory requirements such as HIPAA (USA), GDPR (EU), and, importantly, Kazakhstan’s national regulations (“On Approval of Rules for Collection, Processing, Storage, Protection and Provision of Personal Medical Data by Digital Healthcare Subjects”) (30–32).

From the patient’s perspective, completing questionnaires through a mobile app at their own pace can provide more thoughtful answers and reduce the stress of a clinic visit (33). The ability to remotely monitor the system allows physicians to keep track of chronic patients between visits (34). Finally, the integration of artificial intelligence into MedQuest offers the potential to identify subtle health patterns and develop personalized recommendations, while maintaining data privacy through strong encryption (35).

In light of the growing interest in artificial intelligence for clinical documentation and the limited evidence on its application in functional health classification, this study aimed to rigorously assess the questionnaire completion time and ICF mapping accuracy of the MedQuest mobile application in comparison with conventional paper-based questionnaires. The primary outcomes were: (1) the time required to complete questionnaires during clinical appointments, and (2) the accuracy of AI-generated ICF mapping, evaluated against expert clinician mapping using the quadratic-weighted Kappa statistic. Secondary outcomes included physician satisfaction and the consistency of ICF mapping, assessed through sensitivity, specificity, and concordance matrix analysis.

## Materials and methods

### Study population and data selection

This study was conducted in accordance with the ethical standards of the 2013 Declaration of Helsinki and followed CONSORT guidelines for randomized controlled trials. The protocol was approved by the Local Bioethics Committee of NCJSC Astana Medical University (№7, 27 September 2024) and subsequently registered on ClinicalTrials.gov (NCT07021781, 11 June 2025). All participants provided written informed consent. Adult inpatients (≥18 years) who owned smartphones and were able to operate them independently were recruited at Green Clinic LLC and the National Research Oncology Center LLC, both located in Astana, Kazakhstan. The study population consisted mainly of patients with musculoskeletal disorders, including osteoarthritis of the hip (coxarthrosis) and knee (gonarthrosis), as well as patients with trophic ulcers resulting from atherosclerosis and diabetes mellitus. These conditions were selected because they are common clinical problems that require a comprehensive functional assessment and ICF mapping in rehabilitation medicine. Patients were included if they: (1) had stable medical conditions allowing participation in questionnaire completion, (2) demonstrated basic literacy skills in Russian (the language of the questionnaires), (3) were physically capable of completing the assessment procedures, (4) provided informed consent to participate. Exclusion criteria included severe cognitive or visual impairment, and voluntary withdrawal after protocol explanation. Participants did not receive any financial compensation, and they were free to withdraw from the study at any time without consequence.

### Study design and randomization

This parallel, two-group randomized controlled trial was conducted in two distinct phases. In Phase 1, traditional paper-based questionnaires (control) were compared with the MedQuest mobile app (experimental). A total of 185 participants were randomized into two groups (control n = 92; experimental n = 93) using simple randomization with a computer-generated sequence prepared by an independent statistician (36). Allocation was performed by a blinded coordinator to reduce bias. Recruitment occurred from November 25, 2024, to February 26, 2025. Blinding was not feasible for participants or nursing staff due to the nature of the intervention; however, ICF mapping was independently performed by physicians who were blinded to group assignment and AI outputs.

**Figure 1:**
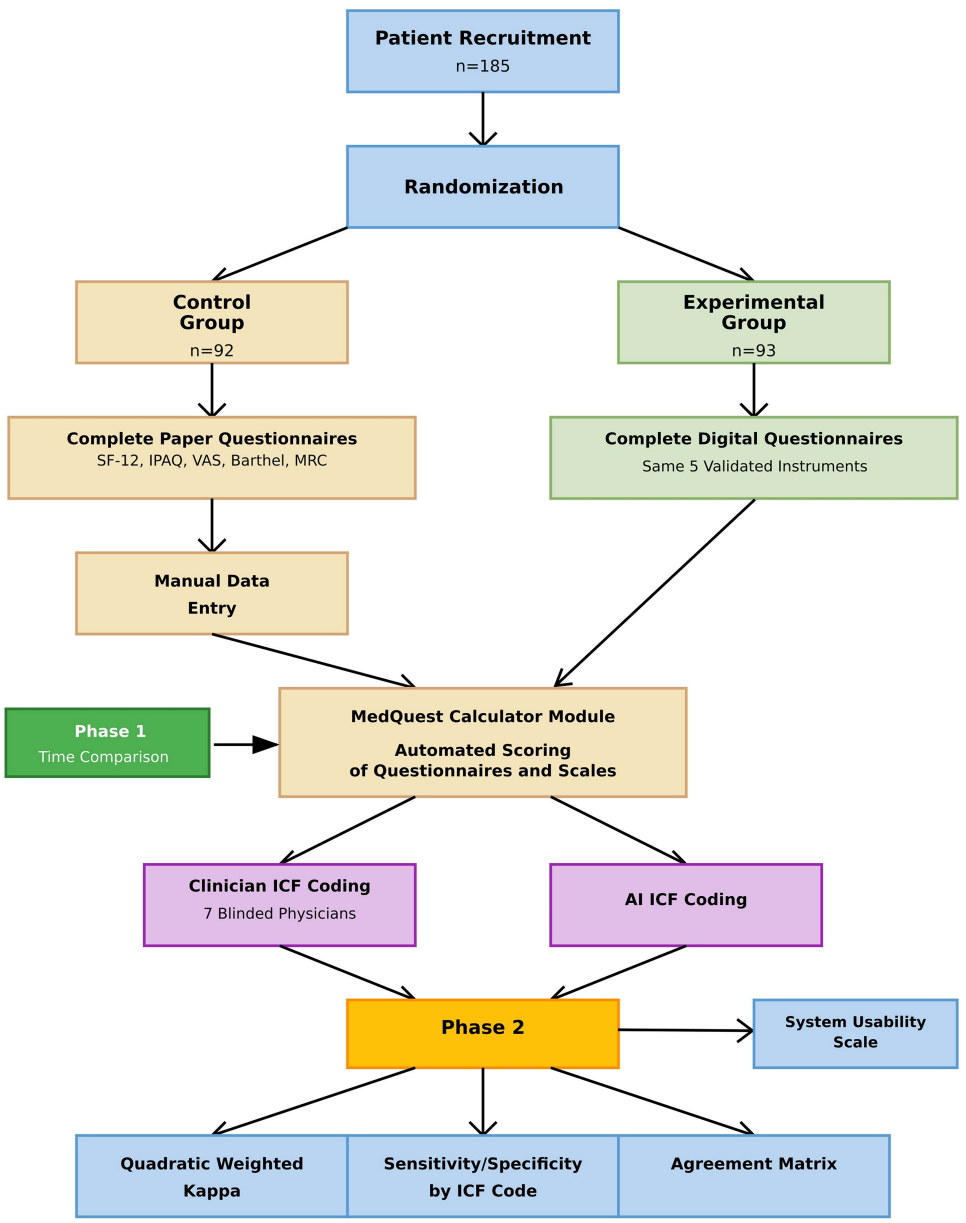
Study flow diagram and data processing workflow.

In Phase 2, results from both groups were combined to compare ICF mappings assigned by clinicians with those generated by artificial intelligence, providing a comprehensive evaluation of AI performance. The overall study structure is illustrated in the flow diagram (Figure 1).

### Interventions

The control group completed validated questionnaires using standardized paper forms, with responses subsequently manually entered by medical staff into the calculator module of the MedQuest app for consistent automated scoring using identical algorithms as the experimental group. The experimental group completed the same questionnaires directly within the MedQuest app, which provided immediate automated scoring and AI-generated ICF mapping. Apart from the completion method, questionnaire content and scoring algorithms were identical between groups, with all results ultimately processed through MedQuest to ensure uniform scoring.

The AI module utilized Anthropic’s Claude 3.5 Sonnet (October 22, 2024), a commercial large language model based on transformer architecture, capable of natural language understanding, analysis, summarization, and dialogue management (37). No additional AI training for ICF was performed; instead, a predefined prompt in Russian guided code generation (see Supplementary files). The system produced structured reports with recommended ICF codes and appropriate qualifiers for comparison with physician-generated codes. Technical infrastructure included Flutter, Dart, Flutterflow for interface creation, Firebase for data storage, and API integration with Claude.

All participants completed identical validated assessments: SF-12 Health Survey (38), International Physical Activity Questionnaire (39), Visual Analog Scale for pain (40), Barthel Index (41), and MRC scale (42). These instruments were selected for their validity and relevance to patient functioning.

### ICF mapping

**Figure 2:**
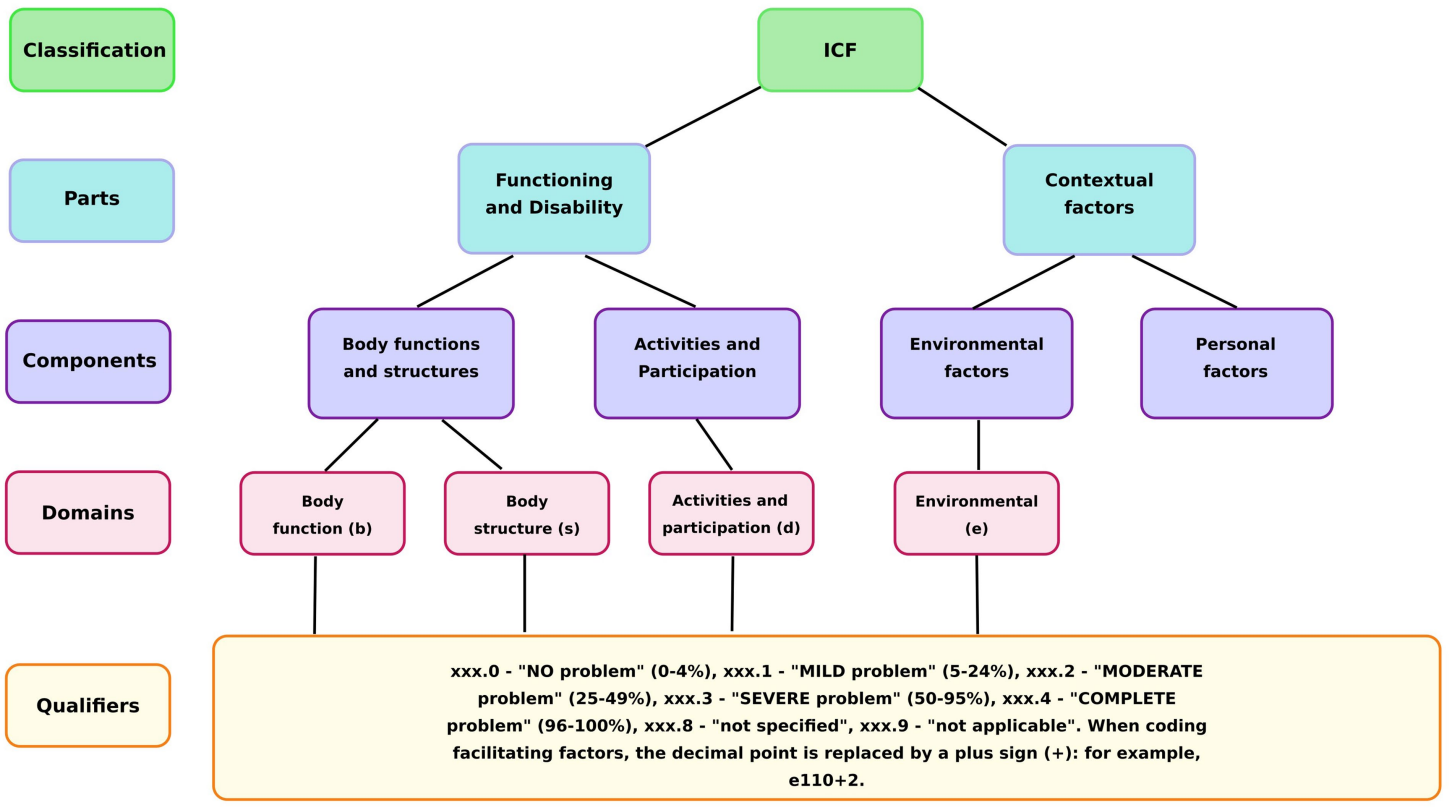
Structure of ICF.

The ICF classification system consists of four main domains, each identified by a specific letter code: Body Functions (b), Body Structures (s), Activities and Participation (d), and Environmental Factors (e). Each ICF code comprises three components: a domain identifier, a numeric code specifying the particular category within that domain, and a qualifier indicating the severity of the problem (Figure 2).

Functional status was mapped using two parallel methods: manual mapping by experienced physicians and automatic mapping by the MedQuest AI system. Manual ICF mapping was performed by seven physicians specializing in rehabilitation medicine. To ensure balanced workload distribution, participants were randomly assigned to physicians using a stratified randomization approach (stratified by study group) that maintained equal distribution across both control and experimental groups. Each physician was assigned approximately 26–27 participants to ensure manageable caseloads while preserving statistical power for inter-rater analysis. Patient assignments were randomized using a computer-generated sequence prepared by the same independent statistician who conducted the initial group randomization, ensuring that each physician evaluated participants from both study groups. To maintain inter-rater consistency, all participating physicians completed a standardized 4-h training session on ICF mapping principles and classification criteria before study initiation. They performed patient interviews, reviewed data from the questionnaires, and systematically matched the information to the appropriate ICF categories and qualifiers. These physician-assigned codes served as the clinical reference standard for subsequent accuracy comparisons. Automatic AI mapping was carried out using the MedQuest application’s Claude 3.5 Sonnet-based AI system (described in Interventions section). Guided by a predefined prompt, the AI analyzed the summary scores from all validated questionnaires to assign the corresponding ICF codes. No predetermined rules were used to link specific questionnaire scores to particular ICF categories; instead, the AI analyzed the aggregate data to determine the appropriate domains and qualifiers.

The conversion of instrument scores into ICF qualifiers (0–4) was based on the severity of the problem. For most scales (SF-12, Barthel Index, IPAQ), the maximum score represented the best health outcome, whereas for the Visual Analog Scale for pain, the maximum score corresponded to the worst outcome. The AI used these principles to assign a qualifier based on the percentage of impairment: “0” for “no problem” (0–4%), “1” for “mild problem” (5–24%), “2” for “moderate problem” (25–49%), “3” for “severe problem” (50–95%), and “4” for “complete problem” (96–100%) (14).

While the assessment tools could have identified other ICF categories, we focused on pre-selected codes that were most relevant to our patient population to evaluate the AI’s performance in the most applicable domains. To ensure objectivity, clinicians were blinded to group assignments and the AI’s output. The codes resulting from both methods were compared *post hoc* to evaluate the AI’s performance and concordance. The study was completed as planned upon reaching the target sample size, with no premature discontinuations.

### Outcomes

The study had two co-primary outcomes reflecting areas of efficiency and accuracy: (1) time to complete questionnaires during the patient encounter and (2) agreement between AI- assigned and clinician- assigned ICF codes, measured using quadratic weighted kappa (*κ* > 0.8 defined near-perfect agreement). For the first primary outcome, the total time taken by each participant to complete all questionnaires was measured in minutes. Time measurement for questionnaire completion was conducted by nursing staff using a standardized protocol. Timing commenced when participants began completing their first questionnaire. For the control group, total time encompassed both patient completion of paper questionnaires and subsequent staff transcription of responses into the scoring app, with timing concluding when transcription was complete. For the experimental group, total time included only patient completion of digital questionnaires within the app, with timing concluding when the patient submitted their final response. Importantly, time spent on subsequent ICF mapping assignments was excluded from questionnaire completion times in both groups to ensure comparable measurement of the core questionnaire completion process.

Secondary outcomes included clinician user satisfaction assessed using the System Usability Scale (SUS) questionnaire (43), AI diagnostic performance metrics (sensitivity, specificity, micro- and macro-averaged metrics), and a confusion matrix to visualize mapping discrepancies, all assessed after data collection. The SUS is a standardized 10-item instrument that captures usability and satisfaction on a 0–100 scale (higher scores indicate better usability) (44); it addresses aspects such as complexity, ease of use, and confidence in using the system.

### Workflow and user interaction within the MedQuest application

**Figure 3:**
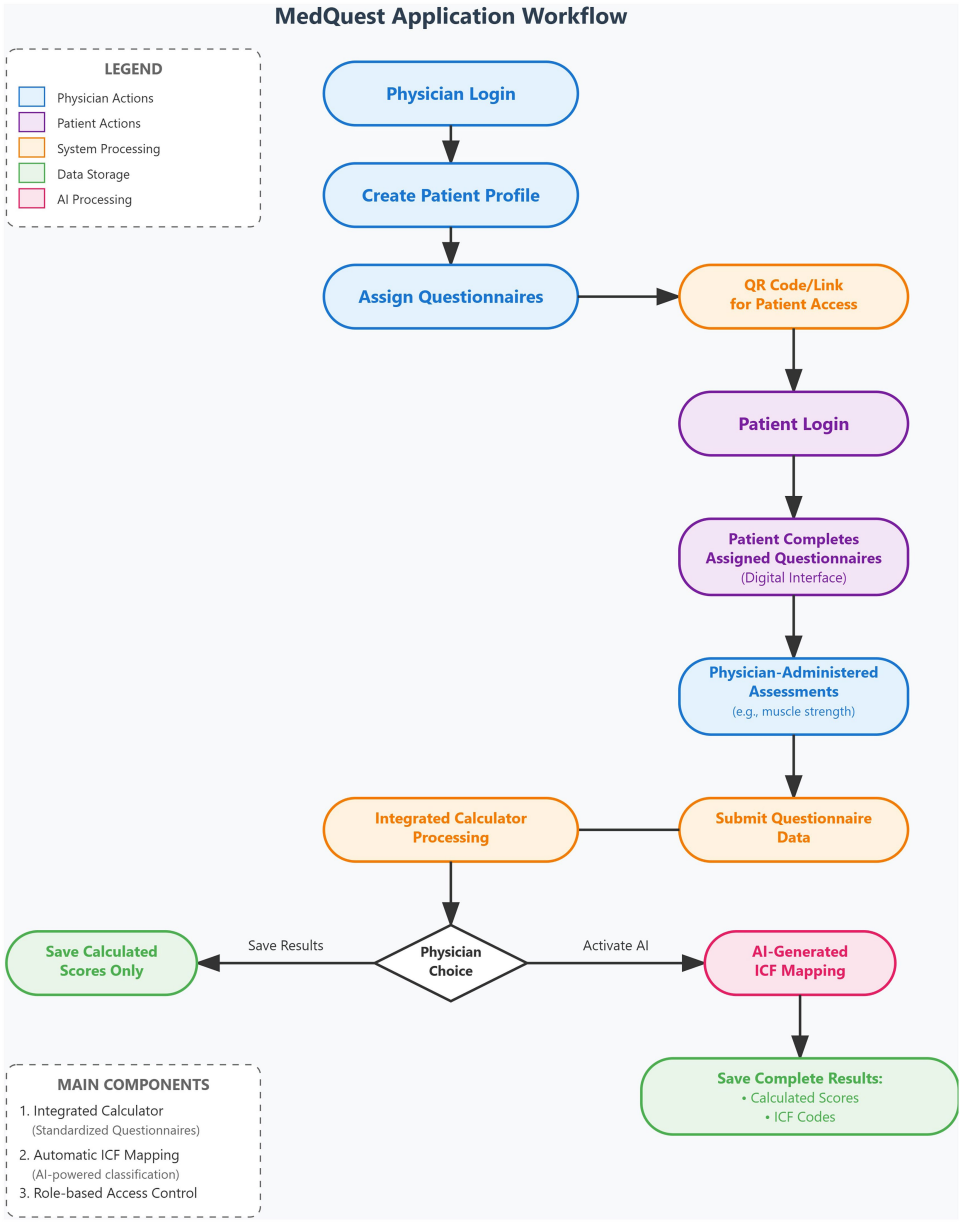
MedQuest application workflow.

The MedQuest mobile application includes two main functional components: an integrated calculator for standardized questionnaires and an automatic ICF mapping system. The application provides a streamlined, role-based workflow for physicians and patients, as illustrated in the complete workflow shown in Figure 3.

**Figure 4:**
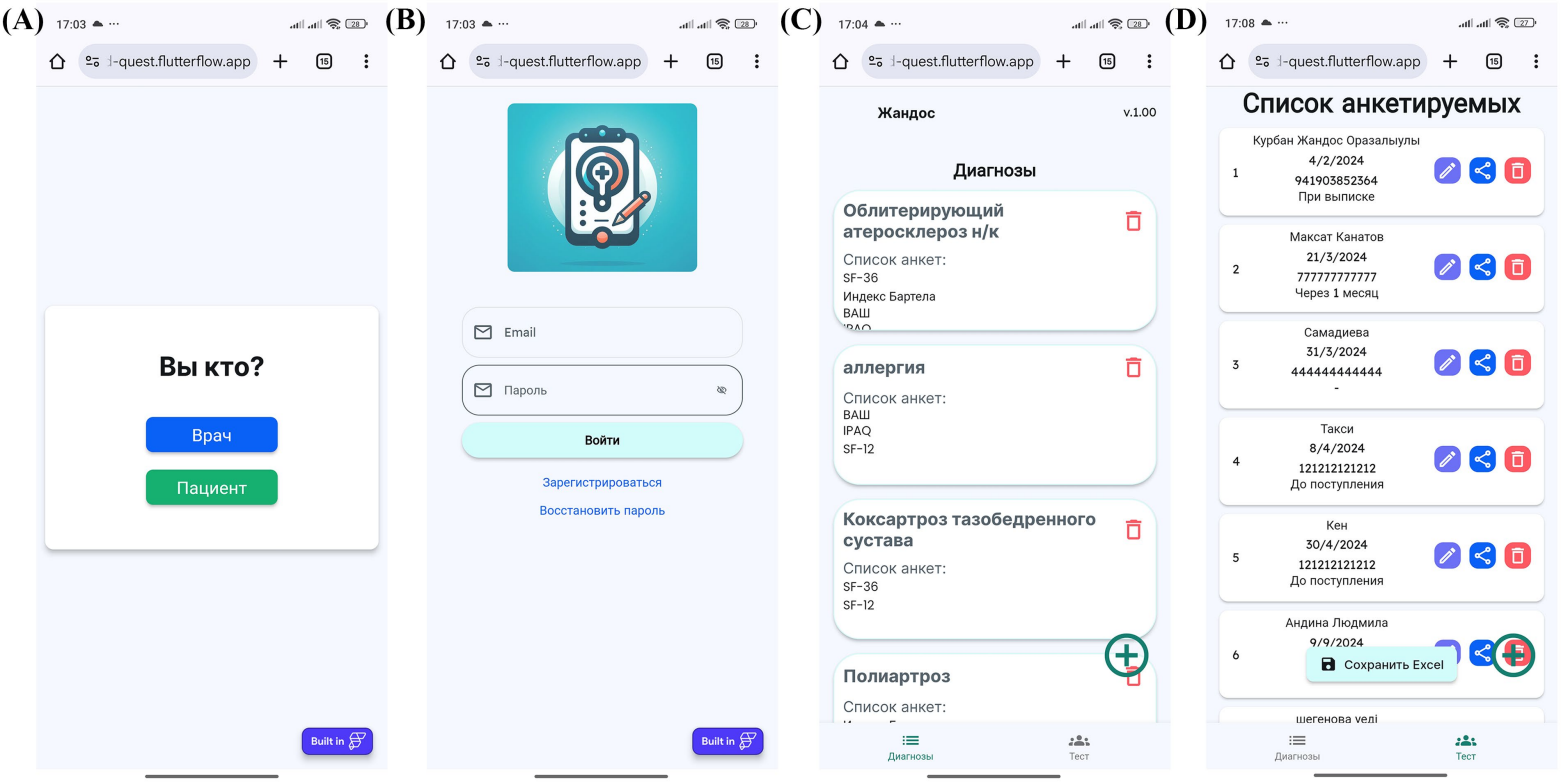
MedQuest mobile application interface for physicians. **(A)** Initial screen for selecting between physician and patient access. **(B)** Physician authorization and login screen. **(C)** Creating a new group of scales for patient assessment. **(D)** Adding a new patient to the system.

**Figure 5:**
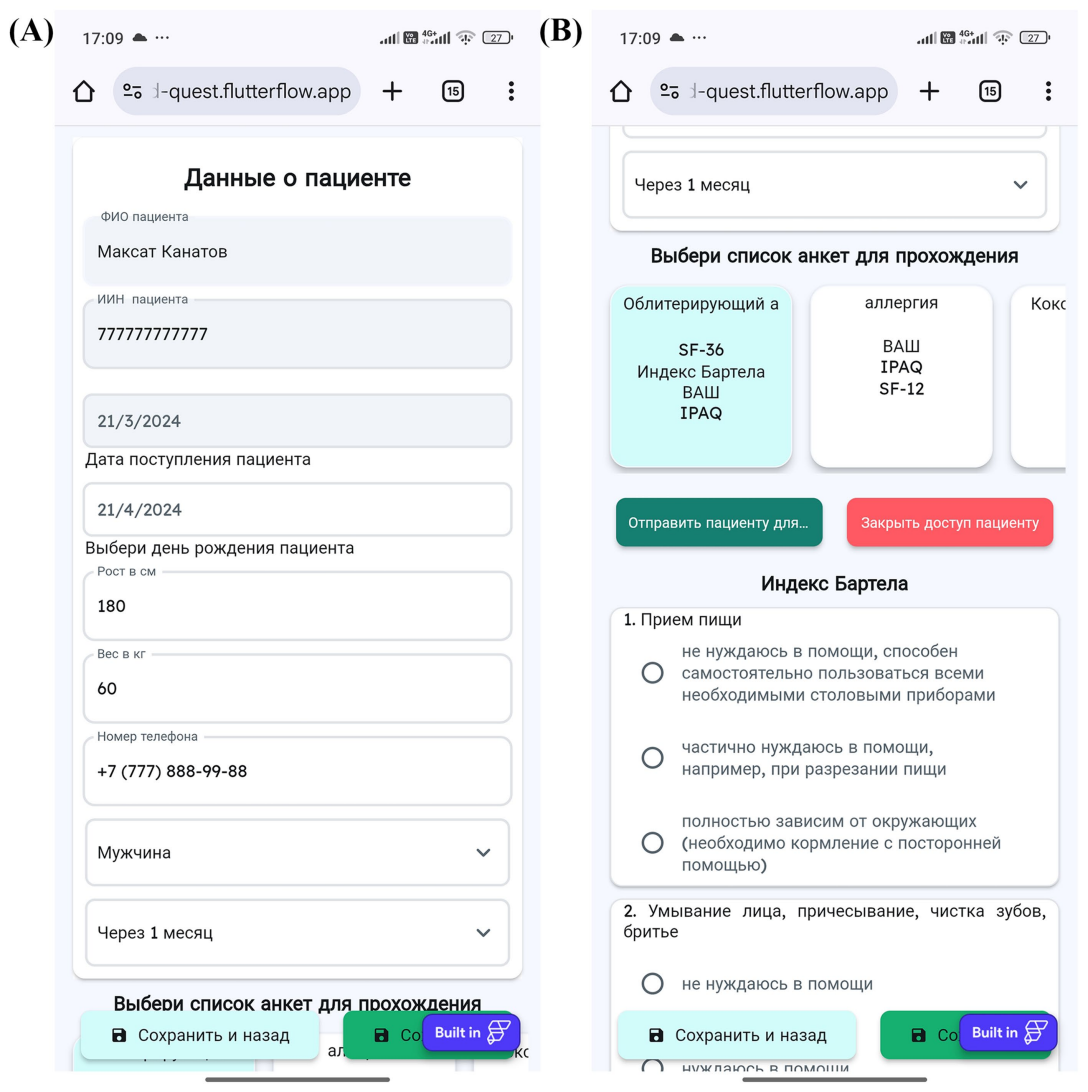
Physician’s workflow for patient data input and access management in MedQuest. **(A)** Detailed patient information entry form. **(B)** Granting the patient access to the assigned questionnaire.

**Figure 6:**
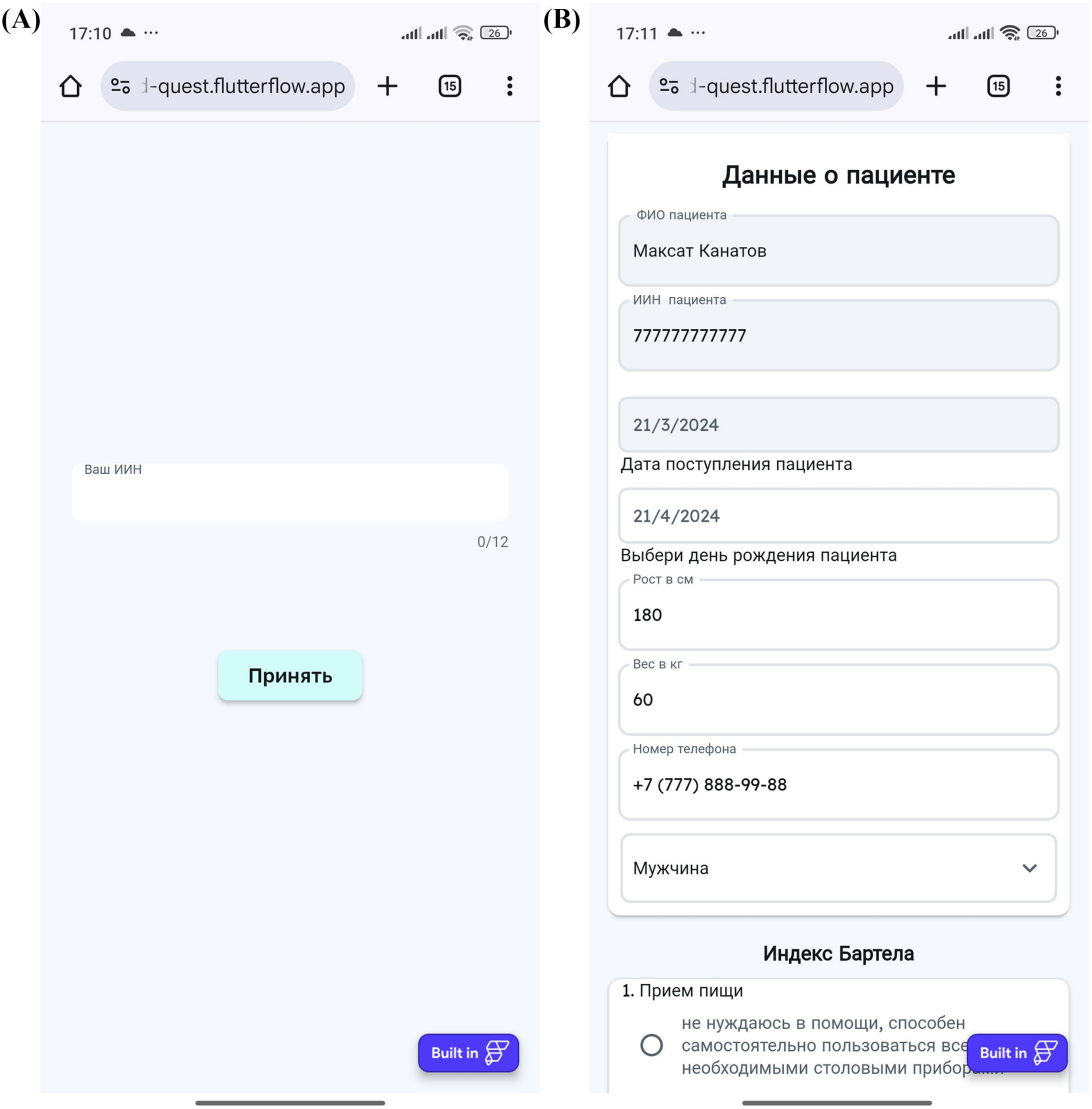
Patient’s interaction with the MedQuest mobile application. **(A)** Patient login screen to access their assigned test. **(B)** Patient interface for completing the assigned questionnaires.

**Figure 7:**
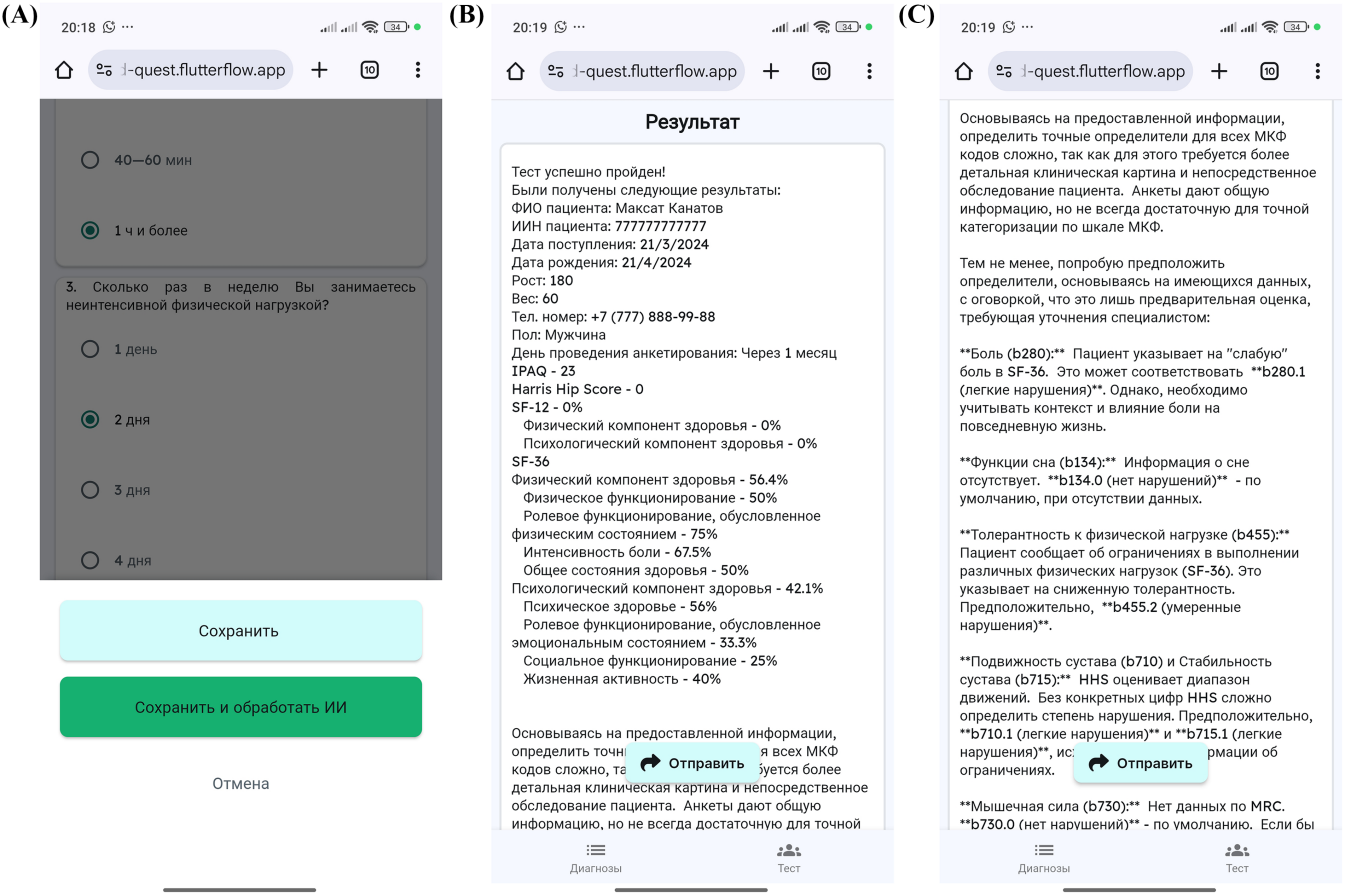
MedQuest mobile application interface for processing and displaying patient data. **(A)** Option to save patient data with or without AI processing. **(B)** Display of standardized scale and questionnaire results. **(C)** AI-generated report showing the recommended ICF codes.

The process begins with physician login and patient profile creation, followed by questionnaire assignment and patient access provision via QR codes or links (Figures 4A–D, 5A,B). Patients then log in and complete their assigned questionnaires through an intuitive digital interface (Figures 6A,B). Upon completion, physicians can either save the calculated scores or activate AI processing to generate structured reports with recommended ICF codes (Figures 7A–C). The application interface and all validated scales and questionnaires are available in Russian.

### Statistical analysis

All statistical analyses were conducted using IBM SPSS Statistics (version 27.0.1.0). The normality of data distribution was assessed using the Kolmogorov–Smirnov test. Quantitative variables with normal distribution are reported as mean ± standard deviation (M ± SD), while non-normally distributed data are expressed as median and interquartile range (Me [Q1–Q3]). Between-group comparisons of demographic and baseline characteristics were performed using the independent-samples *t*-test for normally distributed data and the Mann–Whitney U test for non-normally distributed data. For categorical variables, the Pearson chi-square test was used.

To evaluate agreement between clinician-based and AI-based ICF mapping, Quadratic Weighted Kappa was calculated. This method was selected for its ability to weight larger disagreements more heavily, thus reflecting the clinical impact of rating discrepancies on an ordinal scale. Kappa values were interpreted as follows: *κ* < 0.20—poor agreement, 0.21–0.40—fair, 0.41–0.60—moderate, 0.61–0.80—substantial, and >0.80—almost perfect agreement (45).

In addition to the primary comparisons, prespecified secondary analyses were conducted to explore the AI system’s diagnostic performance. These included calculations of sensitivity, specificity, and both micro- and macro-averaged performance metrics across ICF codes. An agreement matrix was constructed to visualize AI–clinician concordance by functional domain and qualifier level.

All statistical tests were two-tailed, and a *p*-value < 0.05 was considered statistically significant. This multifaceted analytical approach enabled comprehensive assessment of both the MedQuest mobile application’s efficiency and the diagnostic accuracy of the AI-powered ICF mapping module.

### Sample size calculation

Sample size calculations were conducted *a priori* using G*Power (version 3.1.9.7) with the Wilcoxon–Mann–Whitney test selected as the primary statistical test. The effect size (Cohen’s *d* = 0.6) was chosen following Cohen’s established framework for effect size interpretation. Cohen’s seminal work established *d* = 0.6 as representing a medium effect size that is both clinically meaningful and practically detectable (45). This choice reflects our expectation that AI-assisted ICF mapping would demonstrate a clinically significant improvement over traditional methods, while remaining realistic about the magnitude of difference we could reasonably expect to observe. As Sullivan and Feinn emphasize (46), focusing on effect size ensures that we prioritize clinical meaningfulness over mere statistical significance, which is particularly important when validating diagnostic tools that will impact patient care.

The power analysis used the following parameters: two-tailed test, Cohen’s *d* = 0.6, *α* = 0.05, power = 0.95, and 1:1 allocation ratio. This yielded a required sample size of 77 participants per group (154 total). We deliberately chose a conservative approach that balances adequate statistical power with practical feasibility, avoiding the trap of overpowering our study to detect trivial differences that might be statistically significant but clinically irrelevant. The actual recruited sample of 185 participants resulted in an estimated power of 0.97, providing robust capacity to detect meaningful differences while accounting for potential dropouts or missing data.

## Results

### Phase 1

**Figure 8:**
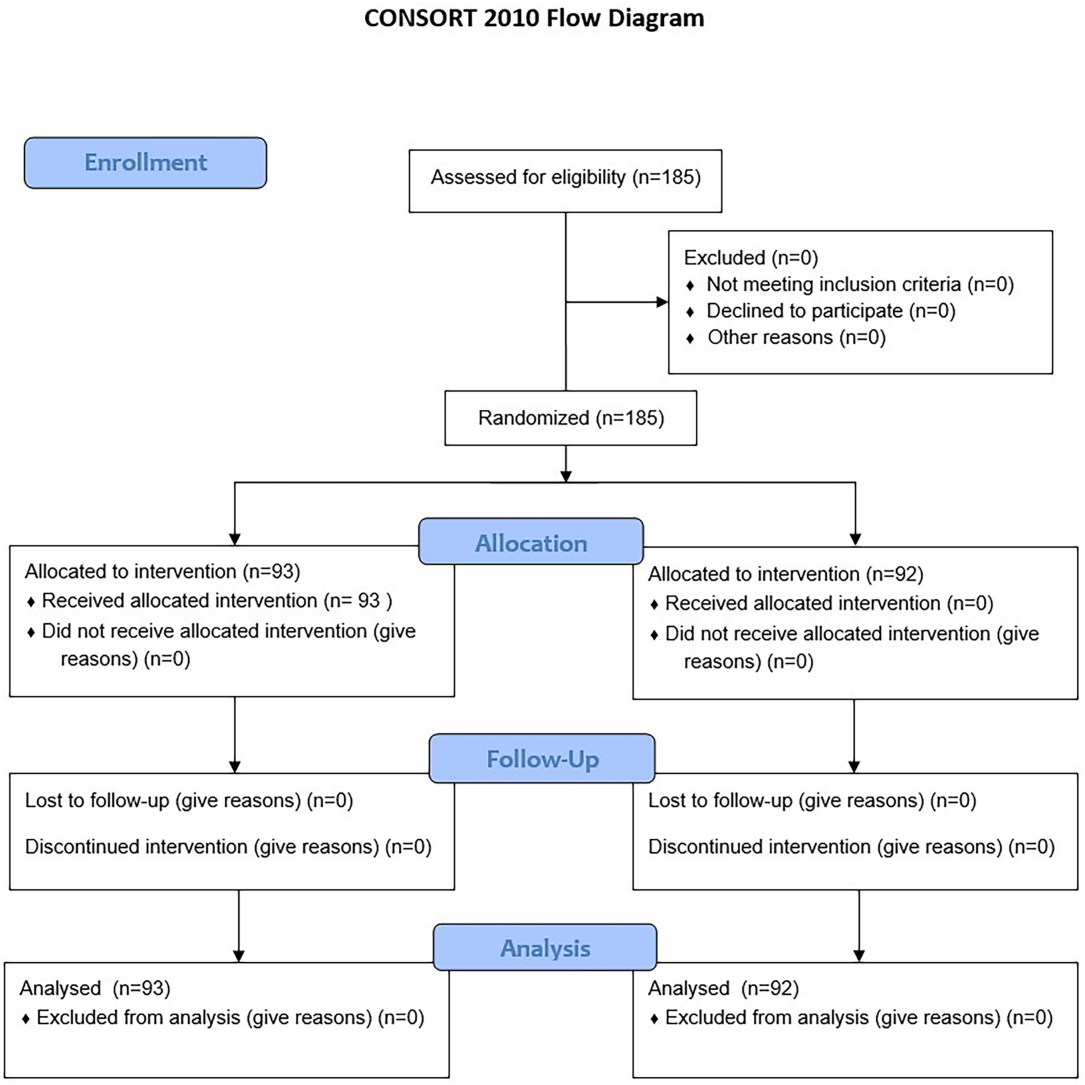
Participant flow chart. The flow chart shows the progression of participants throughout the study.

A total of 185 participants were enrolled in the study and successfully randomized into two groups: a control group (n = 92) and an experimental group (n = 93) (Figure 8). All participants completed the study, and no dropouts or post-randomization exclusions were reported. Baseline demographic characteristics were comparable between the two groups, with no statistically significant differences observed (Table 1). The mean age of participants was 61.77 ± 10.2 years overall, with the control group averaging 61.93 ± 10.5 years and the experimental group 61.61 ± 9.9 years (*p* > 0.05). The median body mass index (BMI) across all participants was 29.7 [IQR: 25.3–34.8]; values were similar between groups, with the control group exhibiting a BMI of 30.1 ± 5.6 and the experimental group 29.5 ± 5.5 (p > 0.05). Gender distribution consisted of 121 males and 64 females, also without significant between-group differences (p > 0.05).

**Table 1:** Comparative characteristics of demographic indicators.

Characteristics	Participants, median (IQR) or mean (SD)			
	All participants (*n* = 185)	Control group (*n* = 92)	Experimental group (*n* = 93)	*p*-value
Socio-demographic characteristics				
Age	61.77 ± 10.2	61.93 ± 10.5	61.61 ± 9.9	0.83
BMI	29.7 [25.3–34.8]	30.1 ± 5.6	29.5 ± 5.5	0.42
Gender				
Male	121	63	58	0.38
Female	64	29	35	

Quantitative data with normal distribution are presented as the mean and standard deviation (mean ± SD); those with non-normal distribution are presented as the median and interquartile range (Me [Q1–Q3]). Mixed formats are shown where appropriate.

Baseline assessment of functional status showed no statistically significant differences between the control and experimental groups (Table 2). Regarding the SF-12 questionnaire, both the physical and mental component scores were comparable: the physical component median was 70 [IQR: 61.25–80] in the control group and 70 [IQR: 60–75] in the experimental group (*p* = 0.22), while the mental component scores were 66.7 [IQR: 55.6–74.1] and 70.4 [IQR: 53.75–79.65], respectively (*p* = 0.40). The Barthel Index medians were 75 [IQR: 70–80] for the control group and 75 [IQR: 65–80] for the experimental group (*p* = 0.13). Pain assessment via the Visual Analog Scale revealed medians of 6 [IQR: 6–7] and 7 [IQR: 5.5–7] in the control and experimental groups, respectively (*p* = 0.97). The International Physical Activity Questionnaire (IPAQ) yielded a median score of 12 [IQR: 10–16] in the control group and a mean of 13.4 [IQR: 10–16] in the experimental group (p = 0.40). Muscle strength, assessed by the MRC scale, was consistent across both groups, with a median score of 4 [IQR: 3–5] (*p* = 0.87).

**Table 2:** Comparative characteristics of functional assessment results in control and experimental groups.

Characteristics		Participants, median [IQR]			
		All participants (*n* = 185)	Control group (*n* = 92)	Experimental group (*n* = 93)	*p*-value
**Results**					
SF-12 questionnaire	Physical component	70 [60–80]	70 [61.25–80]	70 [60–75]	0.22
	Mental component	66.7 [55.6–77.8]	66.7 [55.6–74.1]	70.4 [53.75–79.65]	0.40
	Total component	68.10 [61.7–74.5]	68.1 [61.7–73.95]	70.2 [59.6–76.6]	0.80
Barthel Index		75 [70–80]	75[70–80]	75[65–80]	0.13
Visual Analog Pain Scale		6 [6–7]	6 [6–7]	7 [5.5–7]	0.97
International Physical Activity Questionnaire Short Form (IPAQ)		13 [10–17]	12 [10–16]	13.4 [10–16]	0.40
MRC Muscle Strength Scale		4 [3–5]	4 [3–5]	4 [3–5]	0.87
Time for questionnaire completion		22 [18–28]	28 [26–29]	18 [16–20]	<0.001

Time measured in minutes.

A key finding of the study was the significant difference in the time required to complete the questionnaires. Participants in the experimental group, who utilized the MedQuest mobile application, completed the forms in a median time of 18 min [IQR: 16–20], compared to 28 min [IQR: 26–29] in the control group who used paper-based forms (*p* < 0.001). This reduction represents a 35.7% improvement in completion time, indicating enhanced efficiency associated with the digital application.

### Phase 2

In terms of ICF mapping accuracy, the comparison between clinician-assigned and AI-generated mappings revealed a high level of agreement across the majority of domains (Table 3). Of the 22 domains evaluated, statistically significant differences were found in only three: d410 “Changing body position” (clinicians: 1.76 [IQR: 1–2]; AI: 1.81 [IQR: 1–2]; *p* = 0.04), b280 “Sensation of pain” (clinicians: 2.98 [IQR: 3–3]; AI: 2.94 [IQR: 3–3]; *p* = 0.03), and e310 “Immediate family” (clinicians: 1.22 [IQR: 1–2]; AI: 1.18 [IQR: 1–1]; *p* = 0.02). As shown in Table 3, no differences were found in the remaining domains between the clinician and AI scores (*p* > 0.05). Notably high concordance was observed in domains such as d640 “Doing housework,” d540 “Dressing,” and d430 “Lifting and carrying objects,” where median scores were identical between the clinician and AI ratings. Strong agreement was also evident in functional domains like b455 “Exercise tolerance functions” (clinicians: 2.99 [IQR: 3–3]; AI: 2.98 [IQR: 3–3], *p* = 0.78), b710 “Joint mobility functions” (clinicians: 1.02 [IQR: 0–2]; AI: 1.03 [IQR: 0–2], *p* = 0.71), and b715 “Joint stability functions” (clinicians: 1.04 [IQR: 0–2]; AI: 1.03 [IQR: 0–2], *p* = 0.67).

**Table 3:** Comparative analysis of ICF mapping between clinicians and artificial intelligence system.

	Participants, *n* = 185, median ICF qualifier score [IQR]		
ICF codes	By clinicians	By artificial intelligence	*p*-value
d230 Carrying out daily routine	1.82 [1–2]	1.79 [1–2]	0.18
d410 Changing body position	1.76 [1–2]	1.81 [1–2]	0.04
d420 Transferring oneself	1.82 [1–2]	1.78 [1–2]	0.59
d430 Lifting and carrying objects	1.78 [1–2]	1.78 [1–2]	0.76
d450 Walking	1.61 [1–2]	1.62 [1–2]	0.41
d460 Moving around in different locations	1.58 [1–2]	1.59 [1–2]	0.38
d465 Moving around using equipment	1.62 [1–2]	1.59 [1–2]	0.06
d470 Using transportation	1.59 [1–2]	1.58 [1–2]	0.50
d520 Caring for body parts	1.69 [1–2]	1.67 [1–2]	0.18
d540 Dressing	1.68 [1–2]	1.68 [1–2]	0.87
d640 Doing housework	1.67 [1–2]	1.67 [1–2]	1.00
b134 Sleep functions	1.52 [1–2]	1.54 [1–2]	0.41
b280 Sensation of pain	2.98 [3–3]	2.94 [3–3]	0.03
b455 Exercise tolerance functions	2.99 [3–3]	2.98 [3–3]	0.78
b710 Joint mobility functions	1.02 [0–2]	1.03 [0–2]	0.71
b715 Joint stability functions	1.04 [0–2]	1.03 [0–2]	0.67
b730 Muscle power functions	1.04 [0–2]	1.02 [0–2]	0.15
b770 Gait pattern functions	1.04 [0–2]	1.02 [0–2]	0.29
s770 Additional musculoskeletal structures related to movement	1.42 [1–2]	1.42 [1–2]	0.72
s810 Structure of areas of skin	1.07 [1–1]	1.06 [1–1]	0.38
e310 Immediate family	1.22 [1–2]	1.18 [1–1]	0.02
e540 Transportation services, systems and policies	0.99 [1–1]	1.02 [1–1]	0.11

To further assess the level of agreement between the AI system and clinicians, a Quadratic Weighted Kappa coefficient was calculated, yielding a value of 0.842 (*p* < 0.05), indicating substantial agreement (Table 4). Analysis of the agreement matrix revealed that 80.6% of all scores matched exactly, with 18.7% differing by only one level and discrepancies greater than one level occurring in only 0.6% of cases. The most frequent exact matches were for scores of 2 (1,434 cases, 35.2%) and 1 (1,282 cases, 31.5%). Discrepancies between scores of 1 and 2 (324 cases combined) and between 2 and 3 (212 cases combined) were the most common among adjacent disagreements, further supporting the system’s high reliability in distinguishing between nuanced functional limitations.

**Table 4:** Agreement matrix between clinicians and artificial intelligence in ICF mapping.

	Clinicians					
AI	0	1	2	3	4	Total
0	236 (5.8%)	47 (1.2%)	2 (0.05%)	0 (0.0%)	0 (0.0%)	285
1	129 (3.2%)	1,282 (31.5%)	150 (3.7%)	2 (0.05%)	1 (0.02%)	1,564
2	8 (0.2%)	174 (4.3%)	1,434 (35.2%)	175 (4.3%)	1 (0.02%)	1792
3	0 (0.0%)	11 (0.3%)	37 (0.9%)	326 (8%)	52 (1.3%)	426
4	0 (0.0%)	0 (0.0%)	0 (0.0%)	0 (0.0%)	3 (0.07%)	3
All	373	1,514	1,623	503	57	4,070

Kappa with quadratic weighting—0.842 (*p* < 0.05). Cell shading intensity (darker green) corresponds to higher frequencies of agreement between AI and clinician scores, with shading reflecting the magnitude of counts within the agreement matrix.

To evaluate the AI’s classification performance, sensitivity and specificity were calculated for each ICF score using values extracted from the agreement matrix (Table 5). Sensitivity and specificity values were as follows: Code 0—sensitivity 0.633, specificity 0.987; Code 1—sensitivity 0.847, specificity 0.890; Code 2—sensitivity 0.884, specificity 0.854; Code 3—sensitivity 0.648, specificity 0.972; Code 4—sensitivity 0.053, specificity 1.000. These results suggest that the AI system performs particularly well in distinguishing moderate levels of functional impairment, although it is less sensitive in detecting extreme values such as Code 4.

**Table 5:** Artificial intelligence sensitivity and specificity for each ICF code.

ICF code	Sensitivity (Recall)	Specificity
0	0.633	0.987
1	0.847	0.890
2	0.884	0.854
3	0.648	0.972
4	0.053	1.000

To provide a holistic performance overview, micro- and macro-averaged sensitivity and specificity metrics were computed. The micro-averaged sensitivity was 0.806 and specificity 0.952, indicating strong overall predictive accuracy when weighted by case frequency. The macro-averaged sensitivity was lower, at 0.613, reflecting reduced performance in infrequent categories, while macro-averaged specificity remained high at 0.940.

Finally, the usability of the MedQuest application was evaluated using the System Usability Scale (SUS) among seven clinicians (Table 6). The mean SUS score was 86.8 out of 100, indicating high overall user satisfaction and system usability. The highest-rated aspects included ease of use (4.86/5) and function integration (4.71/5). The intention to use the system frequently and user confidence both received favorable scores (4.57/5). Notably, the negatively worded item assessing system inconsistencies received a mean score of 1.14/5, reflecting a positive perception. Three of the seven clinicians (42.9%) rated the system at or above 97.5, while the lowest individual score was 82.5, supporting consistently high satisfaction across respondents. No adverse events or unintended effects were reported during the course of the study.

**Table 6:** System usability scale (SUS) assessment of the MedQuest mobile application (scores on 5-point scale).

SUS question	Physician 1	Physician 2	Physician 3	Physician 4	Physician 5	Physician 6	Physician 7	Mean score
1. I think that I would like to use this system frequently	5	5	4	4	5	5	4	4.57
2. I found the system unnecessarily complex	1	1	2	1	1	1	1	1.14
3. I thought the system was easy to use	5	5	5	4	5	5	5	4.86
4. I think that I would need the support of a technical person to be able to use this system	2	1	2	2	1	1	1	1.43
5. I found the various functions in this system were well integrated	5	5	4	4	5	5	5	4.71
6. I thought there was too much inconsistency in this system	1	1	1	2	1	1	1	1.14
7. I would imagine that most people would learn to use this system very quickly	5	5	4	4	5	4	5	4.57
8. I found the system very cumbersome to use	1	1	1	2	1	1	1	1.14
9. I felt very confident using the system	5	5	4	4	5	5	4	4.57
10. I needed to learn a lot of things before I could get going with this system	1	1	2	2	1	1	1	1.29
Individual SUS Score (0–100)	97.5	100.0	85.0	82.5	100.0	97.5	95.0	86.8

## Discussion

The MedQuest mobile app substantially reduced the time required for questionnaire completion compared to the paper-based method, streamlining the clinical workflow. In our study, clinicians completed mapping tasks significantly faster with the app, a finding that mirrors results from other mobile documentation tools. For instance, Ehrler et al. reported that a smartphone app reduced nurses’ bedside documentation time by approximately 4.1 min per hour, thereby increasing uninterrupted patient care time (47). Similarly, Poissant et al. found that point-of-care computing saved nurses approximately 24–25% of their documentation time per shift (48). Our usability assessment reinforces these findings. MedQuest received an exceptionally high System Usability Scale (SUS) score, well above the 68-point benchmark for usability and the average score of 77 for digital health apps found in a recent meta-analysis (43). Taken together, the significant time savings and excellent user acceptance (SUS score) suggest MedQuest is well-suited for busy clinical environments. By reducing the administrative burden, the app may allow providers to dedicate more time to direct patient interaction and maintain smoother workflows (47).

The AI’s ICF mapping demonstrated robust agreement with clinician assessments. We measured concordance using Quadratic Weighted Kappa, a standard metric for ordinal classification tasks (49). Our Quadratic Weighted Kappa values were in the “substantial” to “almost perfect” range (i.e., typically >0.75), indicating that the AI’s labels closely matched those of human experts. These results are consistent with other studies of AI–clinician agreement. For example, Faryna et al. reported algorithm–pathologist Quadratic Weighted Kappa values between 0.76 and 0.86 for automated Gleason grading (50). In addition to overall agreement, we evaluated sensitivity and specificity for each ICF category. Performance was highest for common and well-defined codes, declining for rarer or more subjective categories (e.g., those involving personal/social context). The extremely low sensitivity (0.053) for ICF code 4, despite perfect specificity (1.000), likely reflects insufficient data representation for this particular code in our dataset, limiting the statistical meaningfulness of these performance metrics. To assess overall accuracy, we computed both micro- and macro-averaged F1-scores. Both the macro-averaged F1-score (averaging performance across codes) and the micro-averaged F1-score (global accuracy) were strong, and our macro-F1 score is comparable to the 84% achieved by Newman-Griffis et al. in a similar automated ICF mapping task (51). These aggregated measures confirm that the AI performs robustly across most categories while highlighting areas where further improvement is needed. However, we acknowledge that despite the standardized training and stratified assignment procedures, some degree of inter-rater variability among physicians may persist, potentially affecting the comparability of physician-assigned codes used as our reference standard. This assumption of consistent physician coding patterns represents a study limitation that may impact the interpretation of AI-physician agreement metrics, potentially either overestimating or underestimating true AI performance depending on the direction of any systematic differences in physician assessments.

Importantly, we recognize that AI-based tools have strengths in automating routine tasks but limitations in subjective domains. Our analysis revealed the AI’s primary challenges lay with codes requiring nuanced human judgment. Discrepancies in domain d410 (changing basic body position) likely stem from the AI relying solely on questionnaire data, whereas clinicians can directly observe the patient. For domain b280 (sensation of pain), differences may reflect the subjective nature of pain and a clinician’s ability to interpret non-verbal cues inaccessible to the AI. The most significant discrepancies, found in domain e310 (immediate family), likely occur because assessing family dynamics requires understanding complex social interactions that clinicians can interpret more effectively through direct communication. Conversely, the perfect agreement for the ICF code d640, “Doing housework,” (*p* = 1.00) likely stems from both clinicians and the AI relying solely on the objective data from the Barthel Index questionnaire. This functional domain is relatively straightforward to assess, allowing both human evaluators and the AI system to consistently translate questionnaire scores into equivalent ICF codes. Consequently, the identical assessments produced by both methods naturally resulted in no statistical difference between the two approaches. These findings have practical implications. Identifying d410 (changing basic body position) issues enables rehabilitation specialists to more effectively target patient-specific mobility challenges. Information about pain sensation (b280) can indicate whether patients require pain management interventions. Assessment of e310 (immediate family) reveals the need for enhanced family-centered communication strategies. These limitations reflect well-documented concerns about AI’s reduced capacity for empathy, nuanced clinical judgment, and contextual understanding in healthcare settings (52). To address these inherent limitations and maximize AI’s benefits, a human-in-the-loop (HITL) workflow is advisable (53). In such a hybrid model, AI can efficiently generate initial mappings while clinicians retain crucial oversight to review and adjust outputs for ambiguous or complex cases. This collaborative approach, where human judgment is integrated into AI-driven processes, ensures accuracy, mitigates bias, and upholds ethical standards, particularly in high-stakes healthcare applications. Hybrid human–AI teams have consistently outperformed humans or machines working alone. For example, an experimental study of endoscopists found that combining AI suggestions with physician review yielded higher diagnostic accuracy than clinicians working alone (54). In practice, MedQuest could be further enhanced by features that flag confidence levels for certain mappings, referring low-confidence cases to clinicians for expert review. This approach would leverage AI’s speed while preserving human expertise for complex clinical judgments and ensure regulatory compliance.

Study limitations include a restricted geographical scope (two medical centers in one city), absence of long-term follow-up, and limited evaluation to a focused set of ICF codes, and potential inter-rater variability among physicians that may have influenced our reference standard. While all participating physicians received standardized training, we did not conduct formal inter-rater reliability testing, which may have impacted the validity of our clinical reference standard and subsequently influenced agreement metrics between manual and AI-based mapping. Additionally, the sample size was insufficient to provide statistically meaningful weighted kappa values for individual ICF domains and qualifier levels. Technological constraints, such as the need for a smartphone and internet access, may exclude certain populations. The study excluded patients with severe conditions and may have attracted more technologically proficient individuals, introducing a potential selection bias. Furthermore, a cost-effectiveness analysis was not performed, the app’s impact on comprehensive clinical outcomes requires further investigation, and future research should analyze AI performance across a broader range of ICF codes with larger samples to enable domain-specific agreement analysis.

## Conclusion

The integration of the MedQuest mobile application with AI-driven ICF mapping demonstrated notable improvements in clinical workflow efficiency and mapping accuracy. The system significantly reduced questionnaire completion time and showed strong agreement with clinician assessments in most functional domains. While limitations were noted in subjective and complex areas, the findings support the use of AI as an assistive tool under clinician oversight. This hybrid approach may enhance documentation quality and optimize time use in busy healthcare settings, with future research needed to evaluate broader applicability and long-term impact.

## Data Availability

The raw data supporting the conclusions of this article will be made available by the authors, without undue reservation.
