# Supplementary material for: Artificial intelligence–enhanced mapping of the international classification of functioning, disability and health via a mobile app: a randomized controlled trial
Source: Front Public Health. 2025 Aug 5;13:1590401. doi: 10.3389/fpubh.2025.1590401 (PMC12361141; doi:10.3389/fpubh.2025.1590401)
Supplement: Supplementary file 1 [file Data_Sheet_1.zip › Prompt.pdf]

Промпт (in Russian)

Пациенты прошли анкету SF-12(оценки качества жизни пациента) физический компонент, SF-12(оценки качества жизни пациента) психический компонент, SF-12(оценки качества жизни пациента) общий, Индекс Бартела, Визуальная аналоговая шкала боли, IPAQ (International Physical Activity Questionnaire - IPAQ), Оценка силы мышц. SF-12 максимальное значение-100, что означает самый хороший показатель, минимальное значение-0, что означает самый худший показатель. Индекс Бартела максимальное значение-100, что означает самый хороший показатель, минимальное значение-0, что означает самый худший показатель. Визуальная аналоговая шкала боли максимальное значение-10, что означает самый плохой показатель, минимальное значение-0, что означает самый хороший показатель означающий, что боли нет. IPAQ максимальное значение-49, что означает самый хороший показатель, минимальное значение-0, что означает самый худший показатель.

d230 Выполнение повседневного распорядка

d410 Изменение позы тела

d420 Перемещение тела

d430 Поднятие и перенос объектов

d450 Ходьба

d460 Передвижение в различных местах

d465 Передвижение с использованием технических средств

d470 Использование пассажирского транспорта

d520 Уход за частями тела

d530 Физиологические отправления

d540 Одевание

d640 Выполнение работы по дому

b134 Функции сна

b280 Ощущение боли

b455 Функции толерантности к физической нагрузке

b710 Функции подвижности сустава

b715 Функции стабильности сустава

b730 Функции мышечной силы

b770 Функции стереотипа походки

s770 Дополнительные скелетно-мышечные структуры, связанные с движением

s810 Структура кожного покрова

e310 Семья и ближайшие родственники

e540 Транспортные службы, административные системы и политика

Оценку функции, структур организма, активности и участия, факторов окружающей среды проводят с помощью единой шкалы определителей, отражающей тяжесть имеющихся проблем: 0—4% — проблем нет или они ничтожные: его определитель-0; 5—24% — незначительные или легкие: его определитель-1; 24—49% — умеренные (средние или значимые): его определитель-2; 50—95% — тяжелые (значительные): его определитель-3; 96—100% — абсолютные (полные): его определитель-4;

Сформируй список всех МКФ кодов по порядку которые используется у нас с вышеуказанными результатами. Определитель должен отражать состояния пациента. МКФ шифры выбери из вышеуказанных мкф шифров. Кодировка выглядит так: "Шифр" + "." + "Определитель". Например: b280.2

Prompt

Note: This is an English translation of the original Russian prompt used in the study.

Patients completed the questionnaire SF-12 (patient quality of life assessment) physical component, SF-12 (patient quality of life assessment) mental component, SF-12 (patient quality of life assessment) general, Barthel Index, Visual Analog Pain Scale, IPAQ (International Physical Activity Questionnaire - IPAQ), Muscle strength assessment. SF-12 maximum value-100, which means the best indicator, minimum value-0, which means the worst indicator. Barthel Index maximum value-100, which means the best indicator, minimum value-0, which means the worst indicator. Visual Analog Pain Scale maximum value-10, which means the worst indicator, minimum value-0, which means the best indicator meaning that there is no pain. IPAQ maximum value-49, which means the best indicator, minimum value-0, which means the worst indicator.

d230 Carrying out daily routine

d410 Changing body position

d420 Transferring oneself

d430 Lifting and carrying objects

d450 Walking

d460 Moving around in different locations

d465 Moving around using equipment

d470 Using transportation

d520 Caring for body parts

d540 Dressing

d640 Doing housework

b134 Sleep functions

b280 Sensation of pain

b455 Exercise tolerance functions

b710 Joint mobility functions

b715 Joint stability functions

b730 Muscle power functions

b770 Gait pattern functions

s770 Additional musculoskeletal structures related to movement

s810 Structure of areas of skin

e310 Immediate family

e540 Transportation services, systems and policies

Assessment of body functions, body structures, activities and participation, environmental factors is conducted using a unified qualifier scale reflecting the severity of existing problems: 0-4% - no problems or negligible problems: its qualifier-0; 5-24% - mild or slight problems: its qualifier-1; 25-49% - moderate (medium or significant) problems: its qualifier-2; 50-95% - severe (considerable) problems: its qualifier-3; 96-100% - complete (total) problems: its qualifier-4;

Generate a list of all ICF codes in order that are used by us with the above-mentioned results. The qualifier should reflect the patient's condition. Select ICF codes from the above-mentioned ICF codes. The coding looks like this: "Code" + "." + "Qualifier". For example: b280.2
