# Supplementary figures and images for: Artificial intelligence–enhanced mapping of the international classification of functioning, disability and health via a mobile app: a randomized controlled trial
Source: Front Public Health. 2025 Aug 5;13:1590401. doi: 10.3389/fpubh.2025.1590401 (PMC12361141; doi:10.3389/fpubh.2025.1590401)

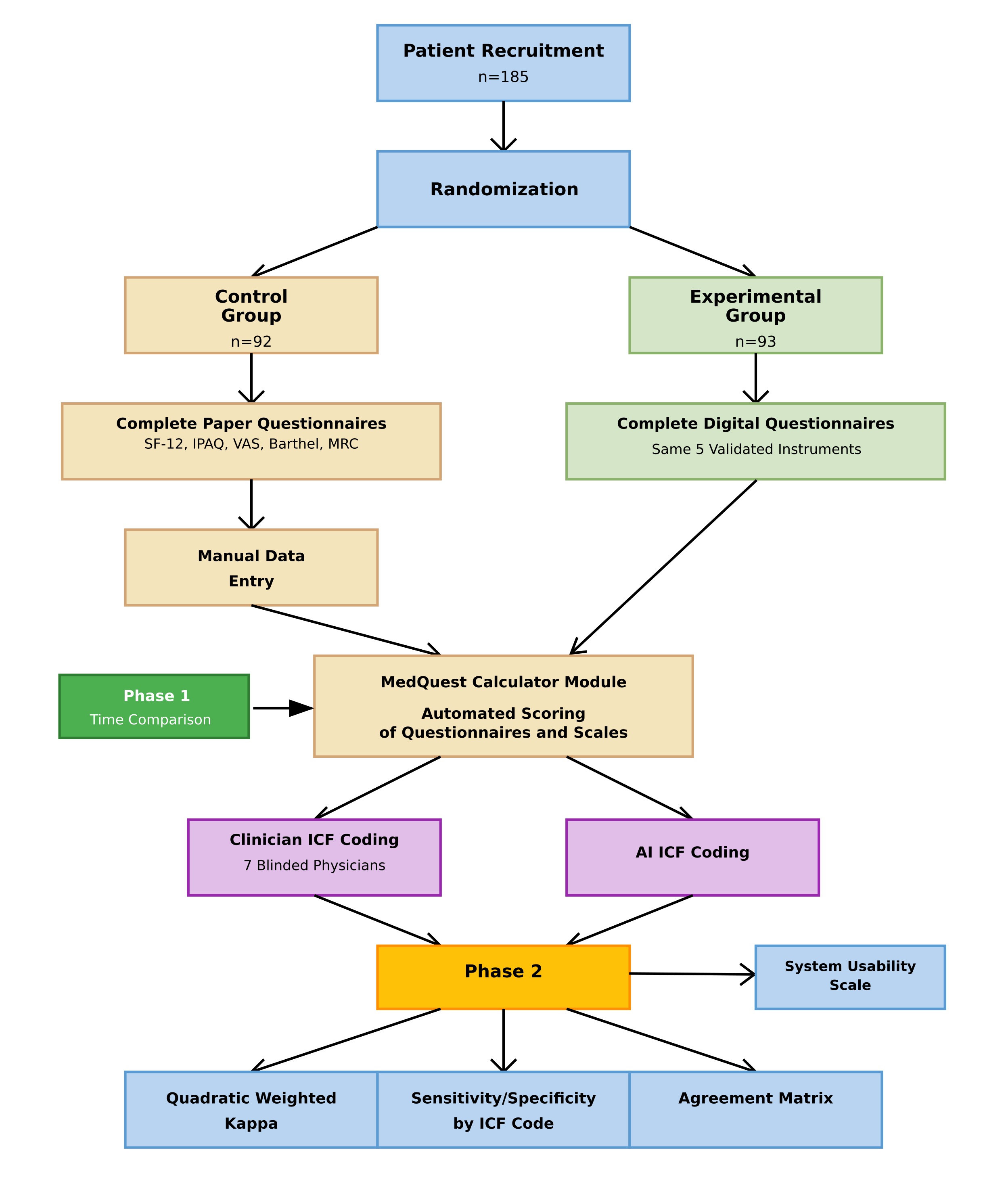

Supplement: Supplementary file 1 [file Data_Sheet_1.zip › Figure 1.jpg]

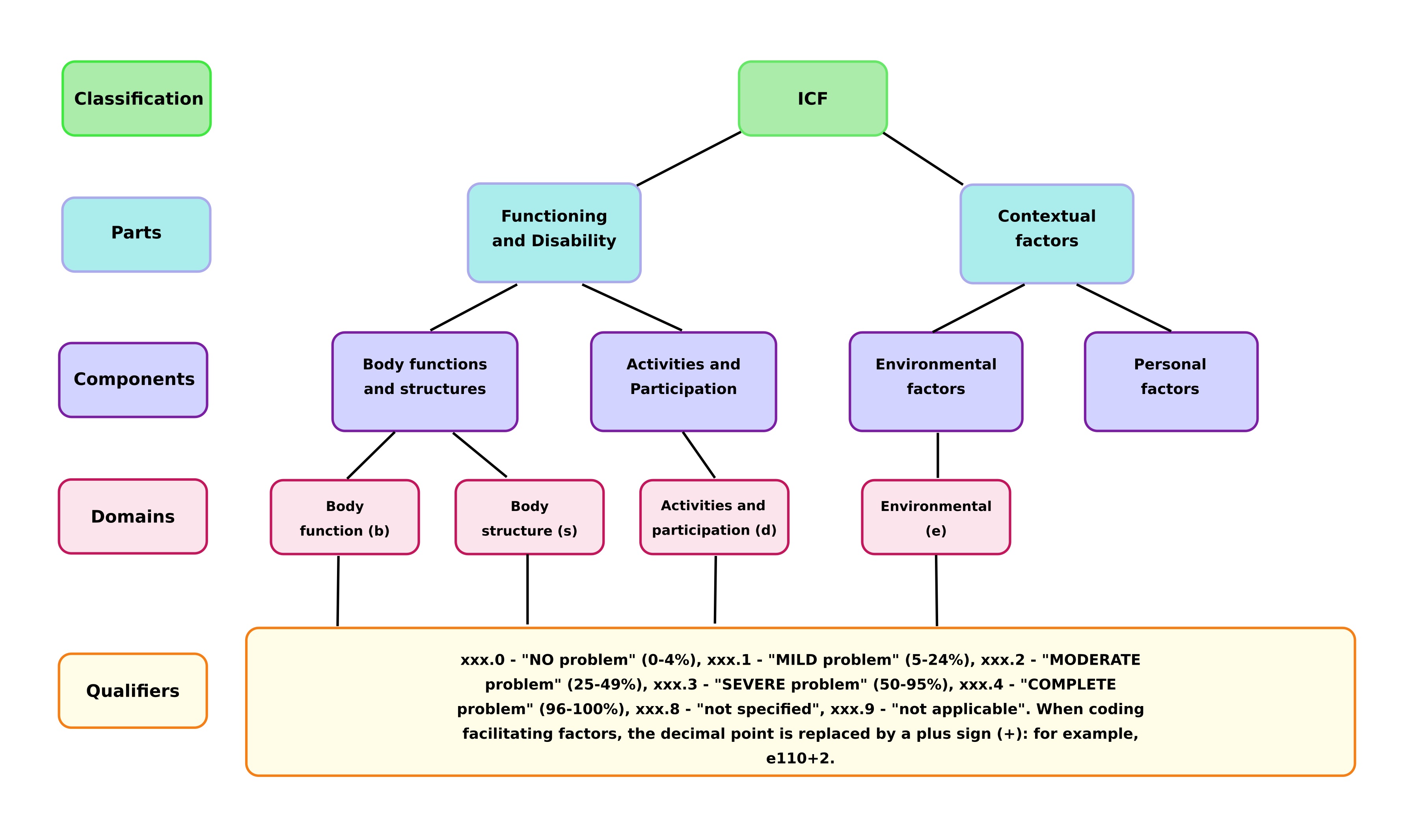

Supplement: Supplementary file 1 [file Data_Sheet_1.zip › Figure 2.jpg]

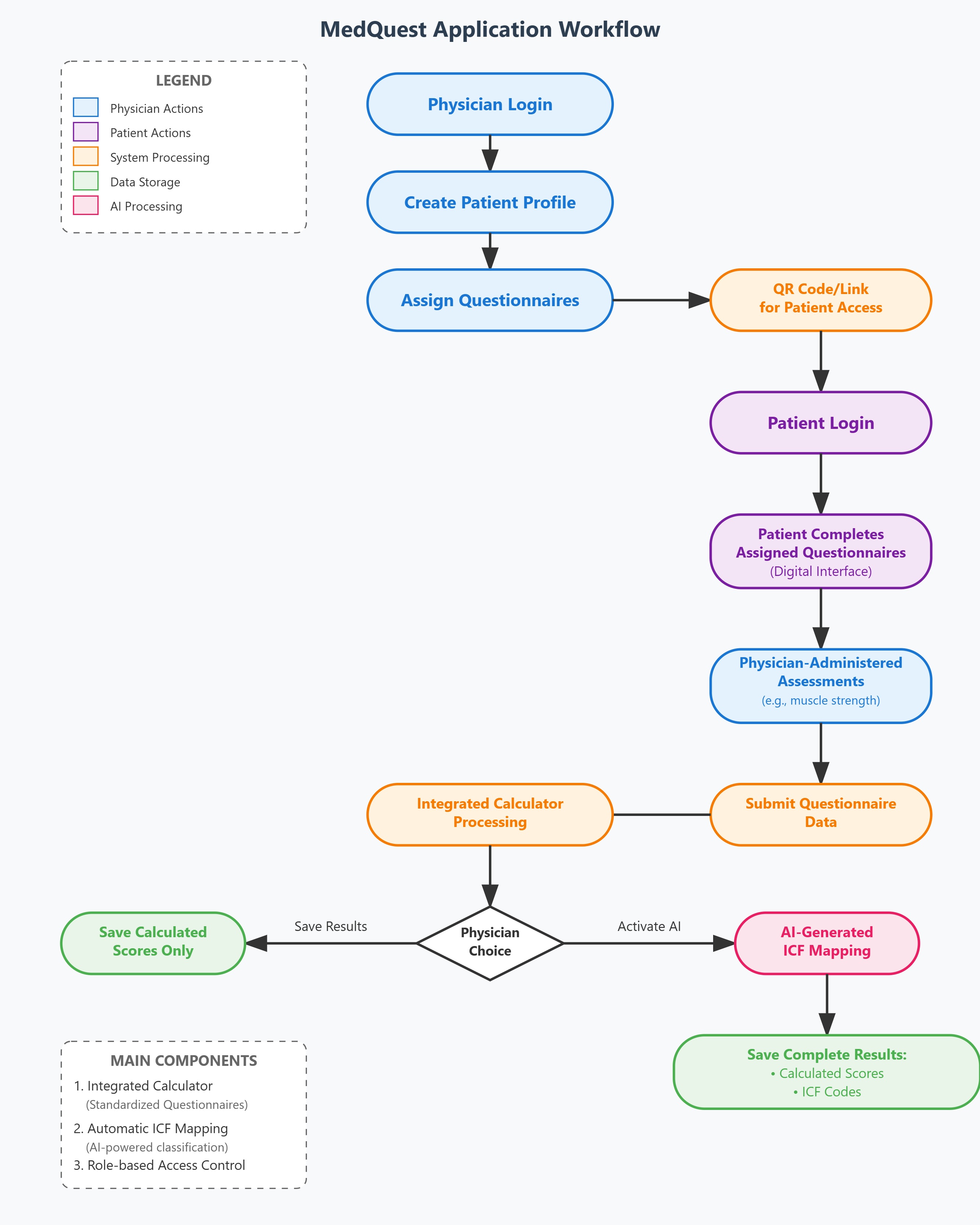

Supplement: Supplementary file 1 [file Data_Sheet_1.zip › Figure 3.jpg]

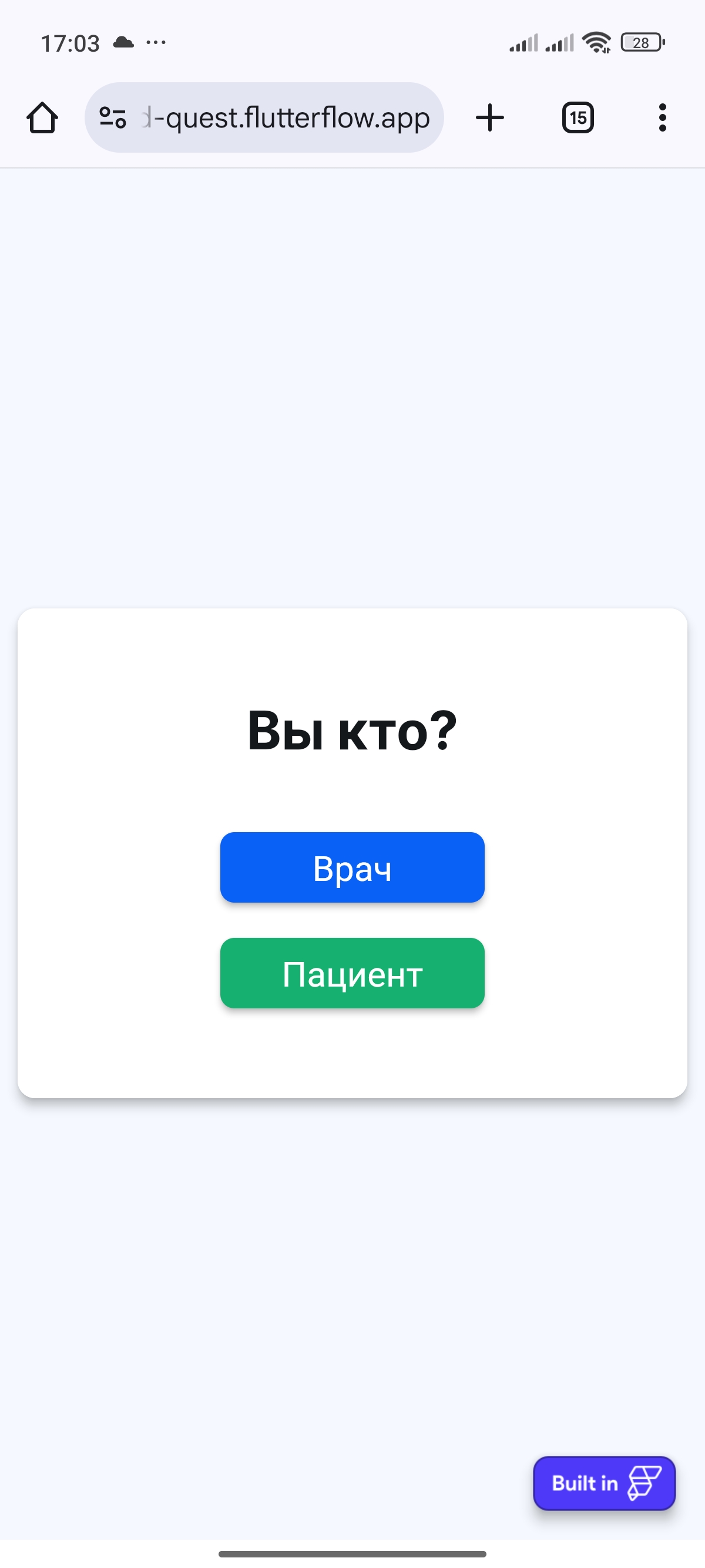

Supplement: Supplementary file 1 [file Data_Sheet_1.zip › Figure 4 (A).jpg]

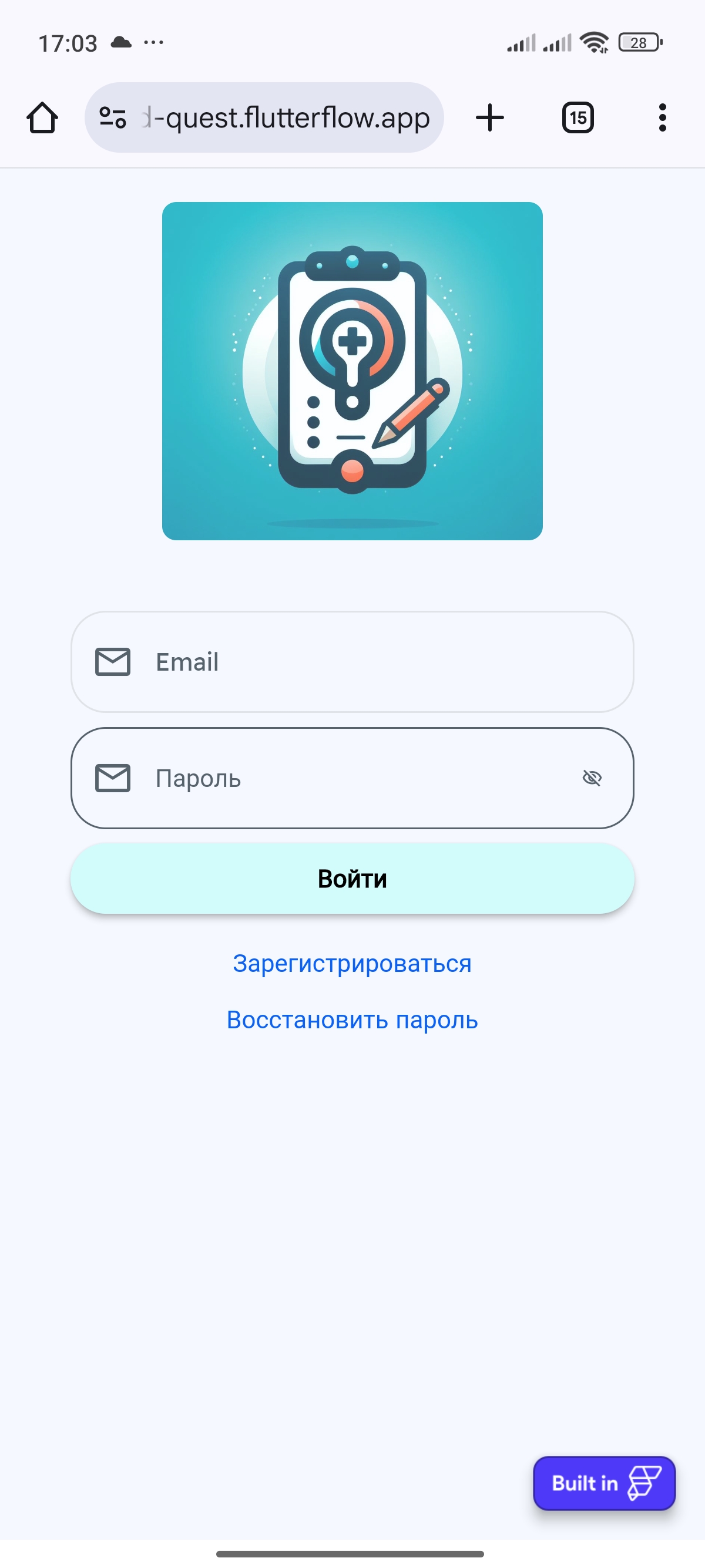

Supplement: Supplementary file 1 [file Data_Sheet_1.zip › Figure 4 (B).jpg]

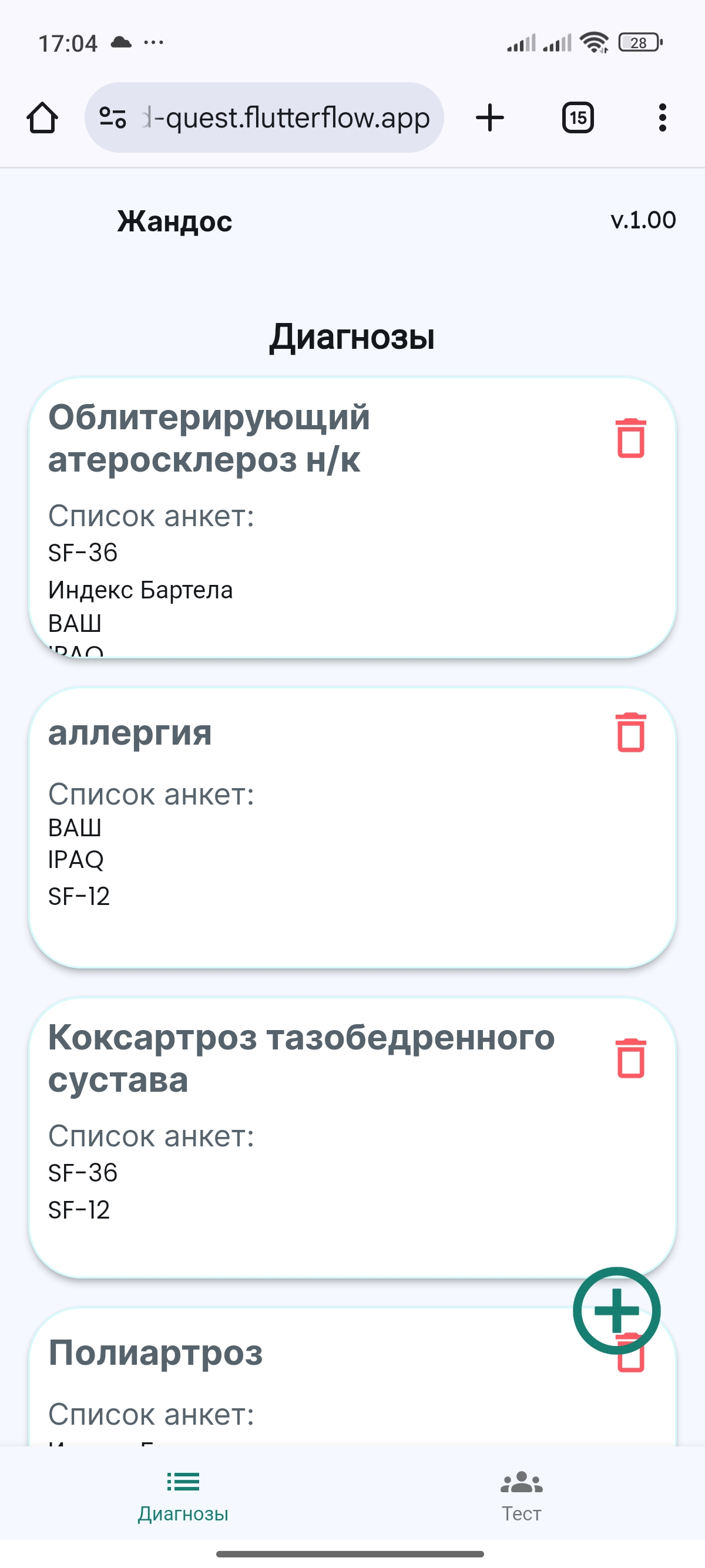

Supplement: Supplementary file 1 [file Data_Sheet_1.zip › Figure 4 (C).jpg]

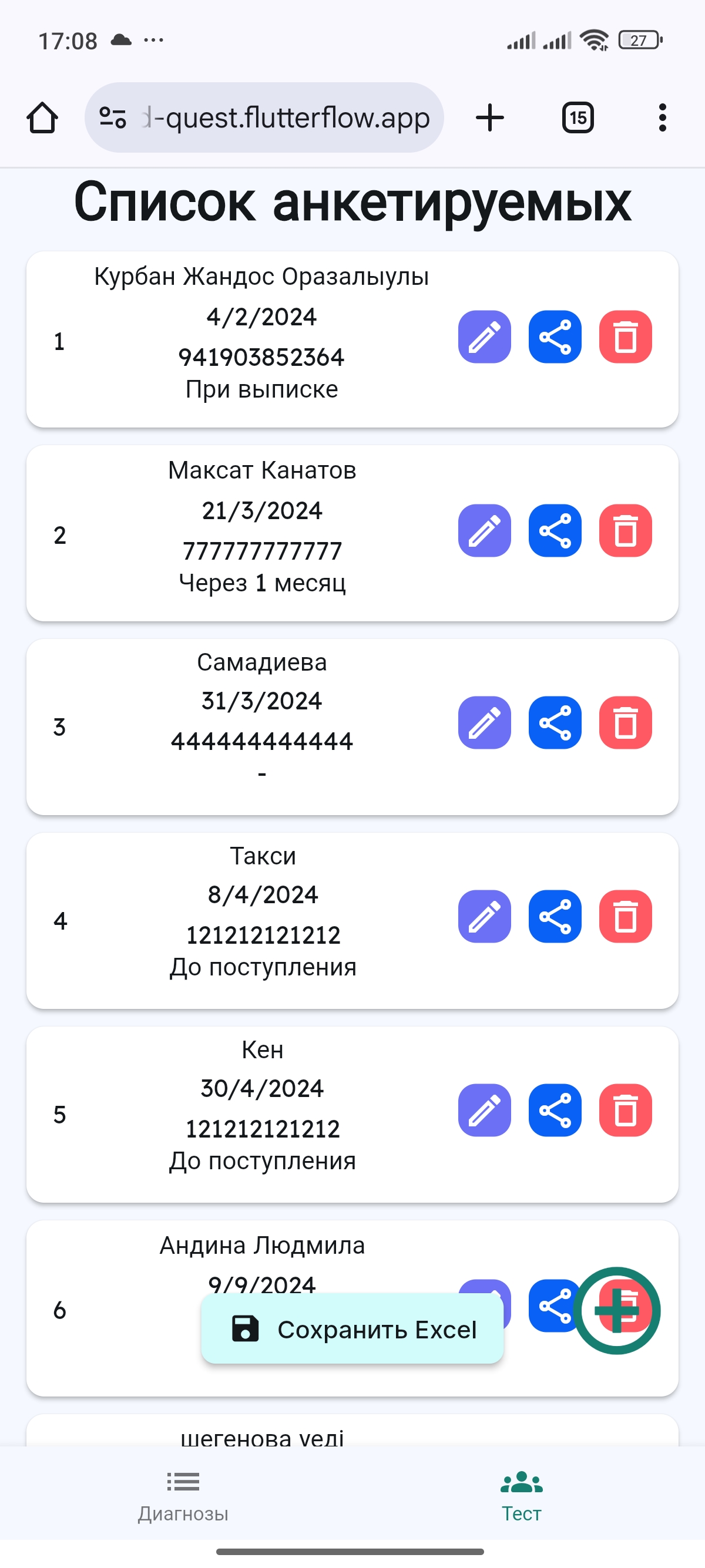

Supplement: Supplementary file 1 [file Data_Sheet_1.zip › Figure 4 (D).jpg]

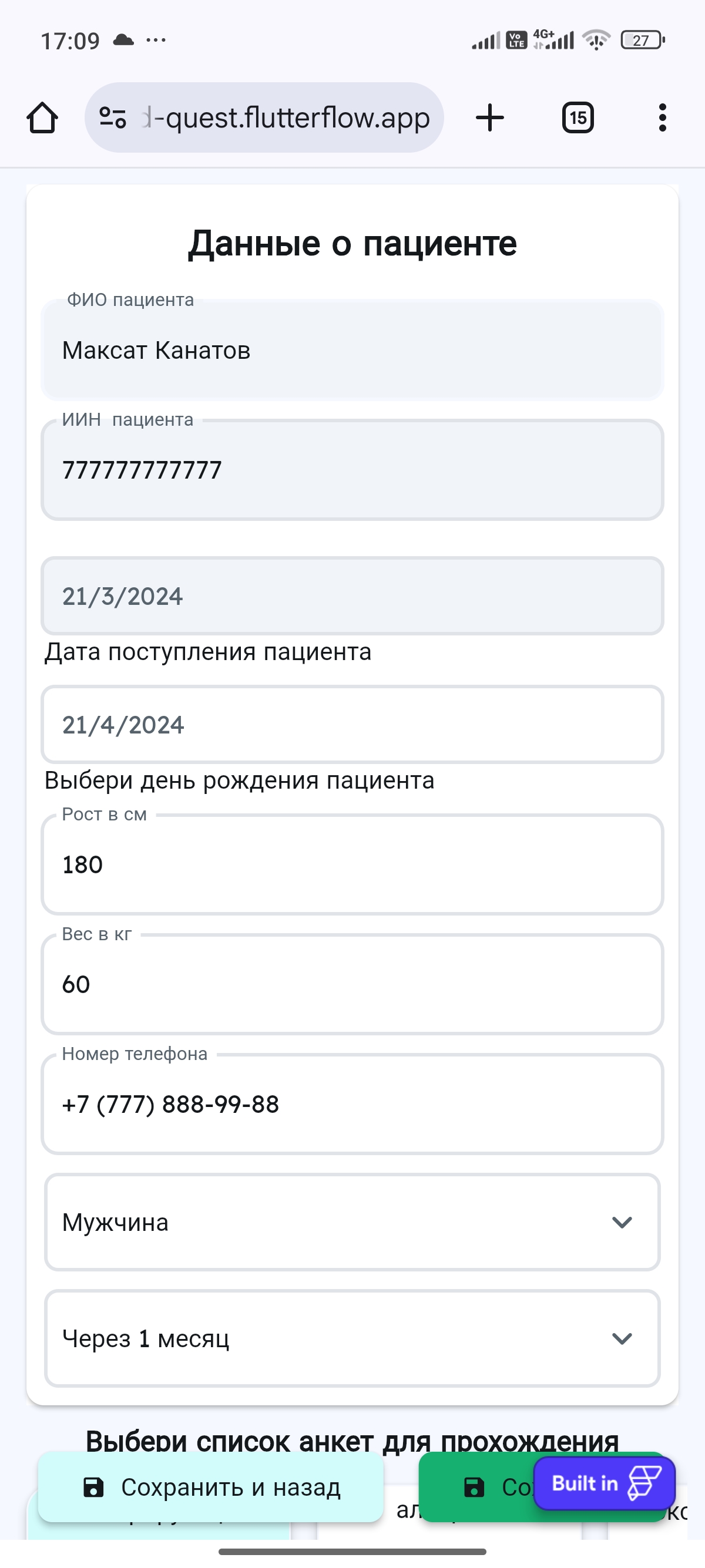

Supplement: Supplementary file 1 [file Data_Sheet_1.zip › Figure 5 (A).jpg]

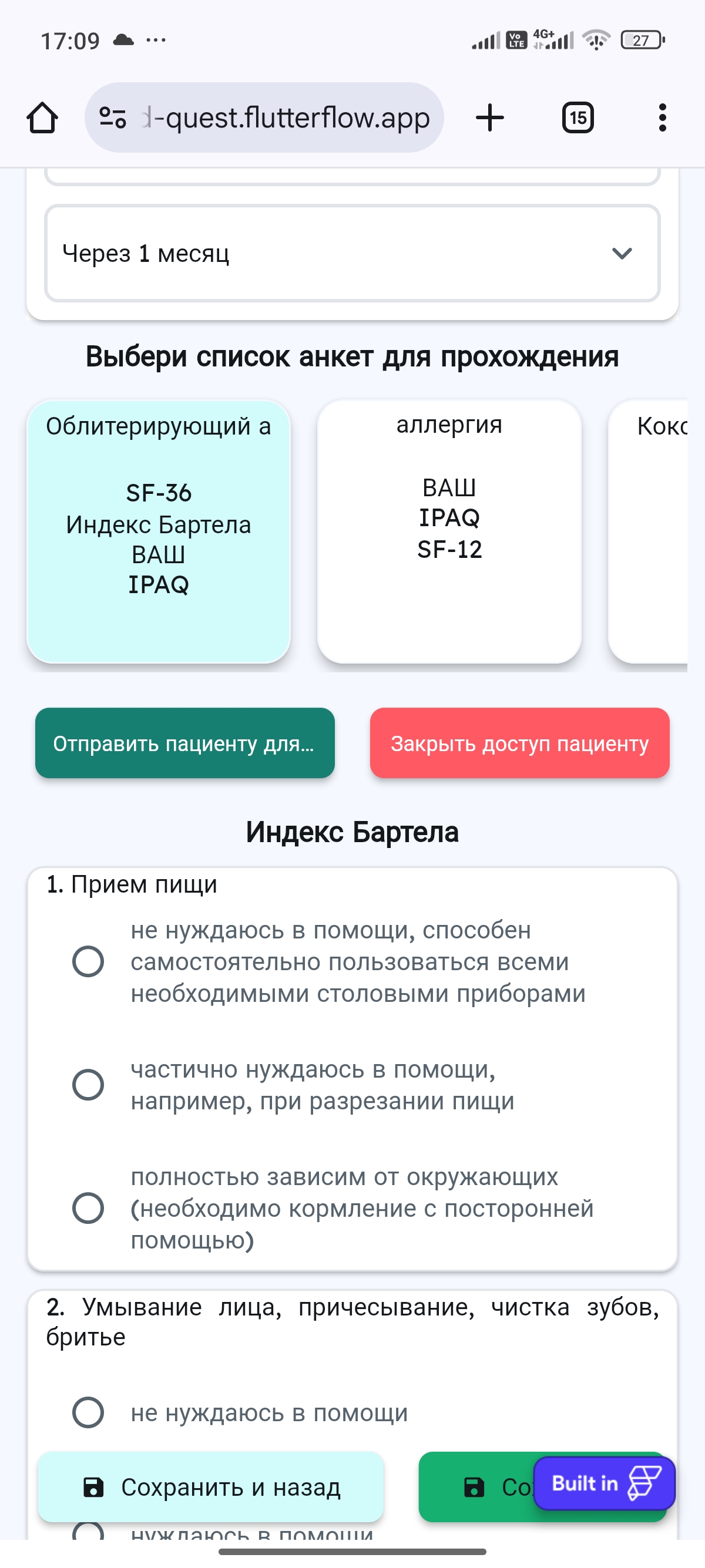

Supplement: Supplementary file 1 [file Data_Sheet_1.zip › Figure 5 (B).jpg]

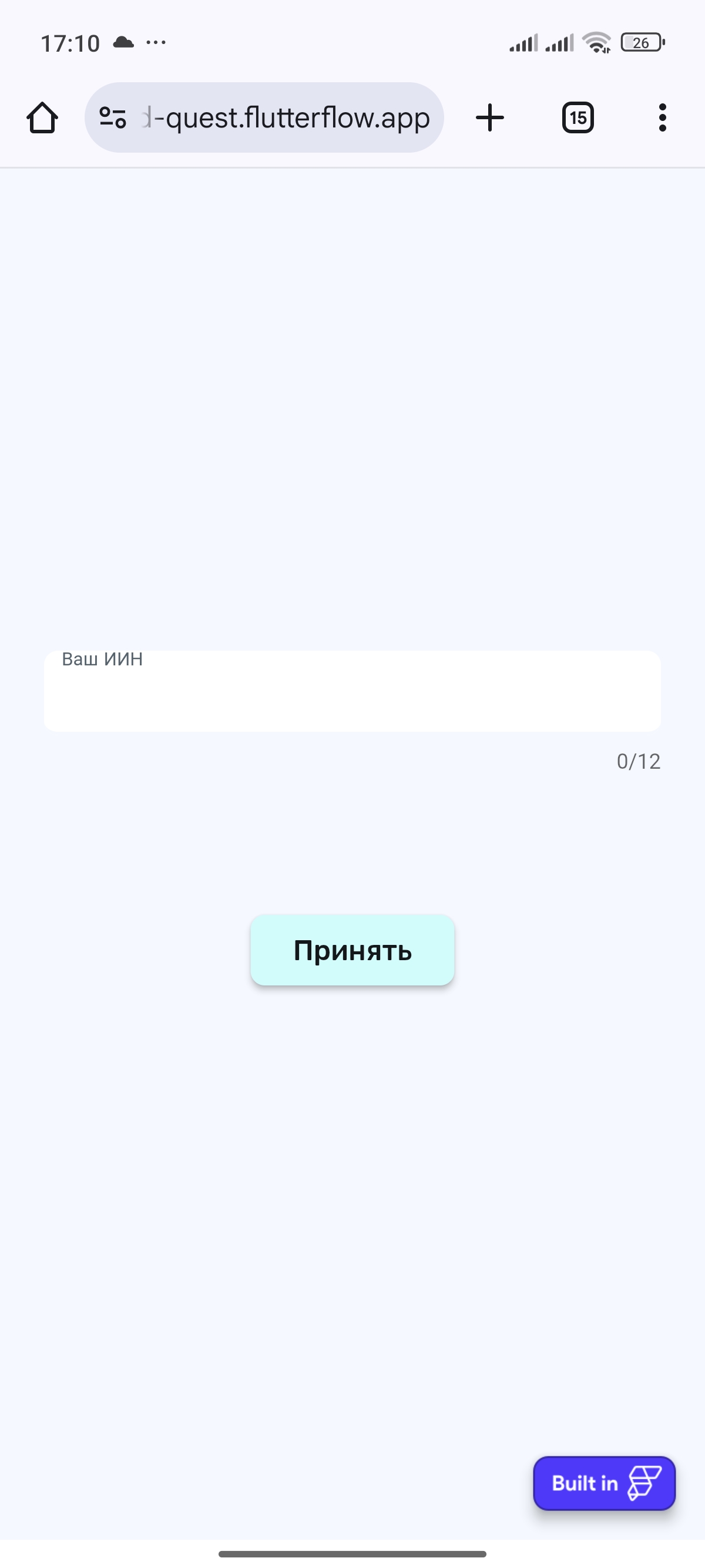

Supplement: Supplementary file 1 [file Data_Sheet_1.zip › Figure 6 (A).jpg]

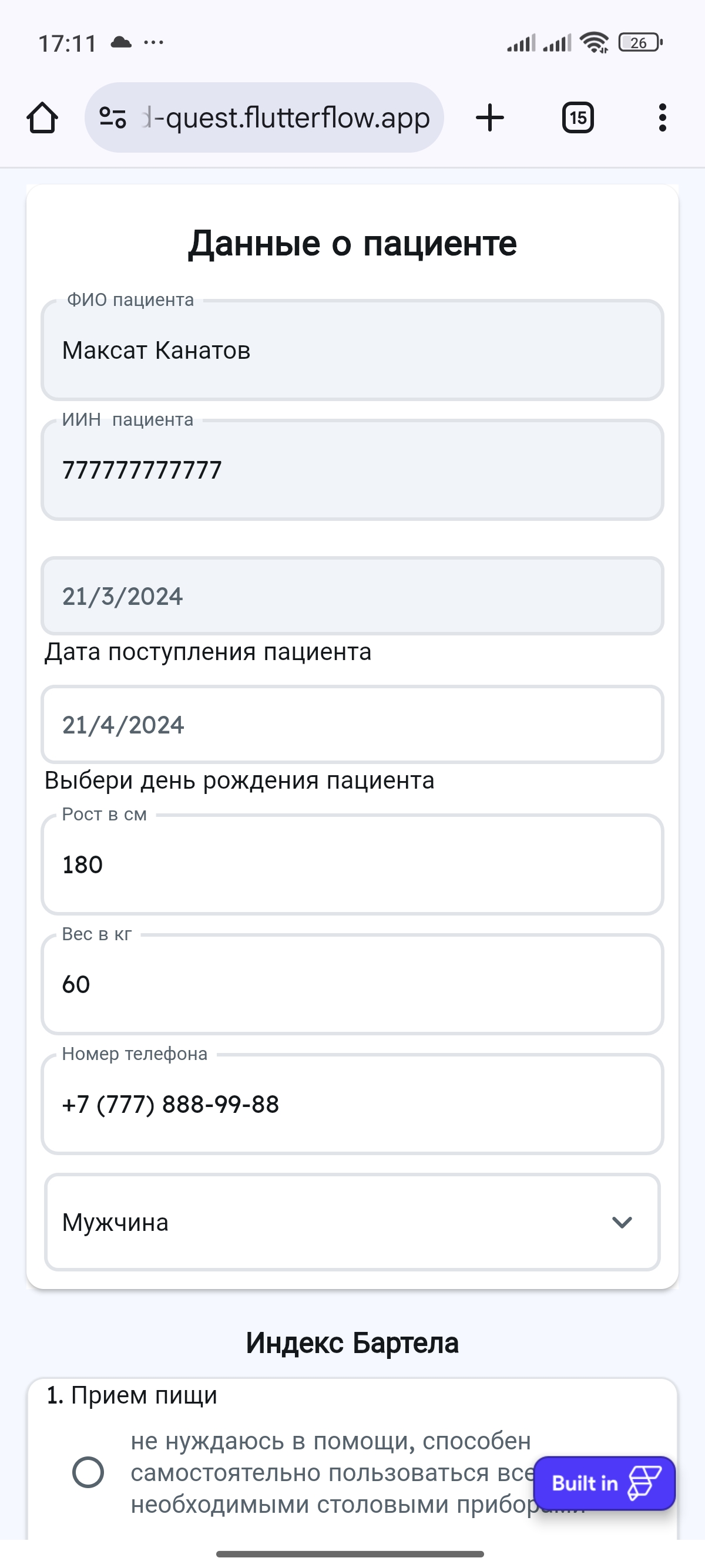

Supplement: Supplementary file 1 [file Data_Sheet_1.zip › Figure 6 (B).jpg]

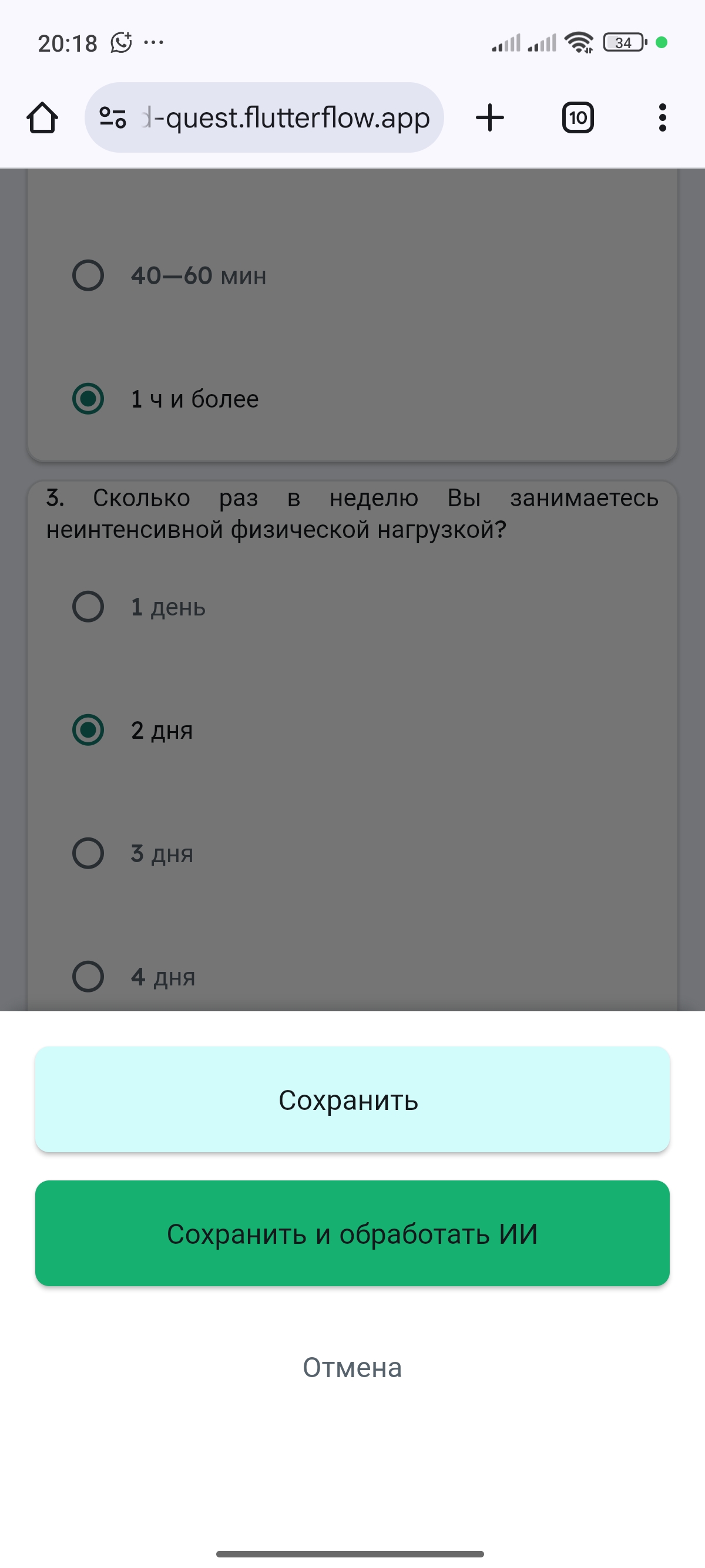

Supplement: Supplementary file 1 [file Data_Sheet_1.zip › Figure 7 (A).jpg]

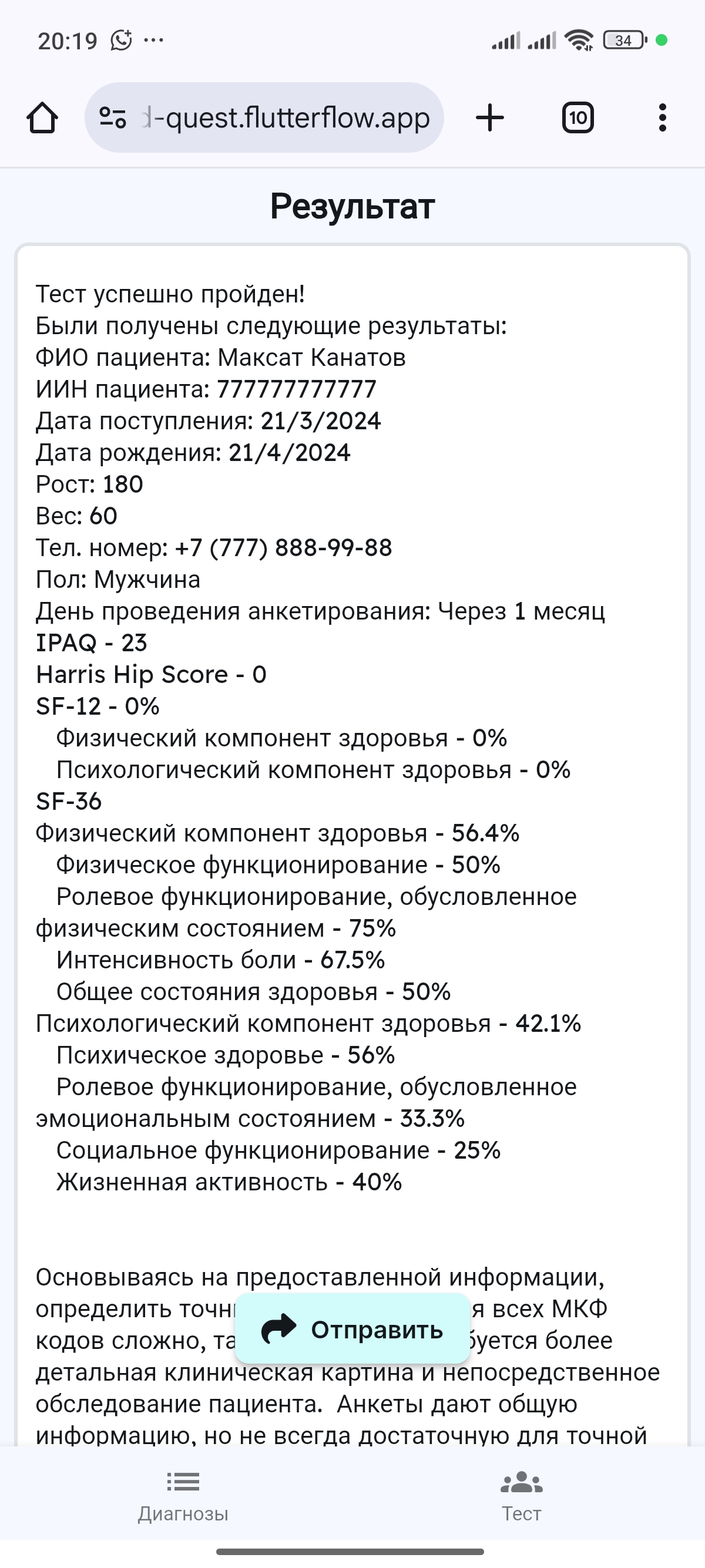

Supplement: Supplementary file 1 [file Data_Sheet_1.zip › Figure 7 (B).jpg]

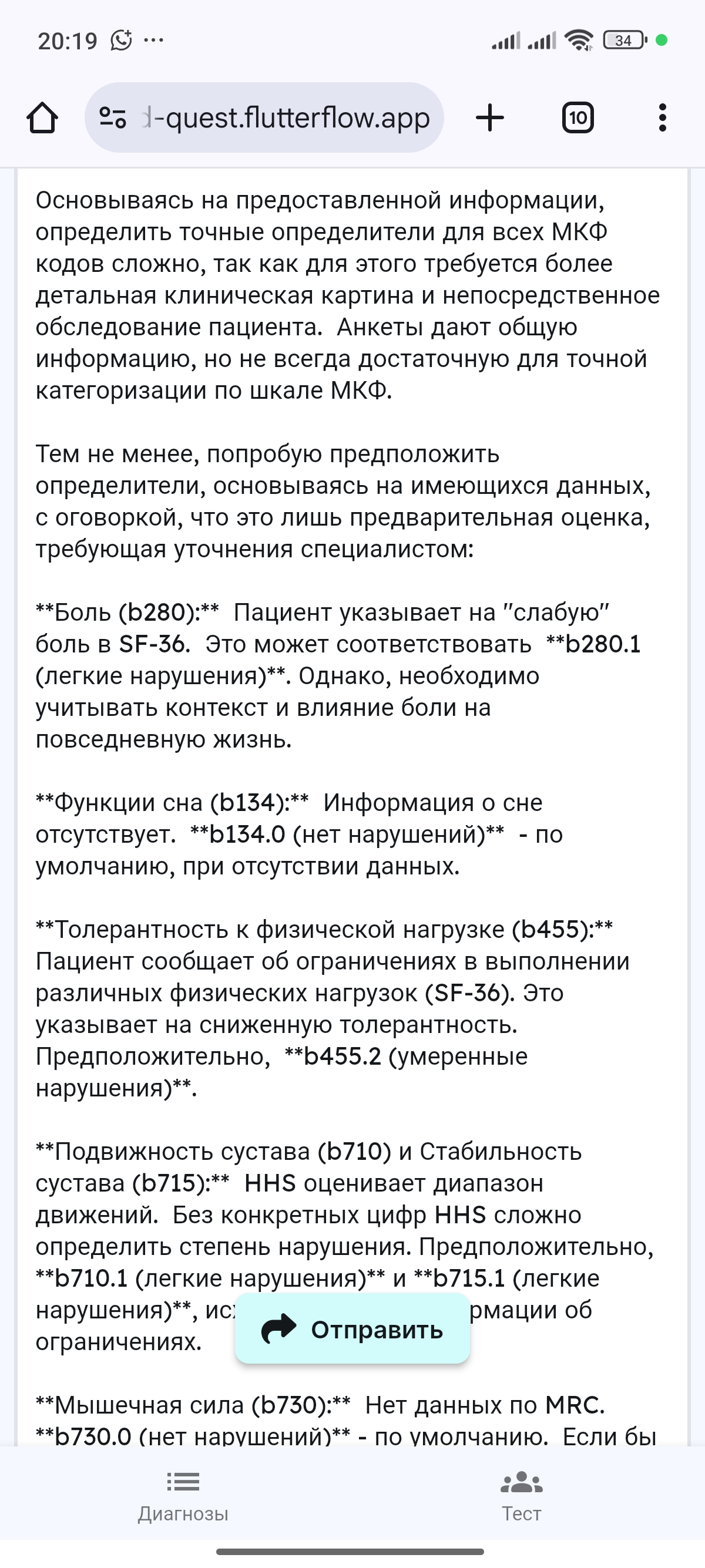

Supplement: Supplementary file 1 [file Data_Sheet_1.zip › Figure 7 (C).jpg]

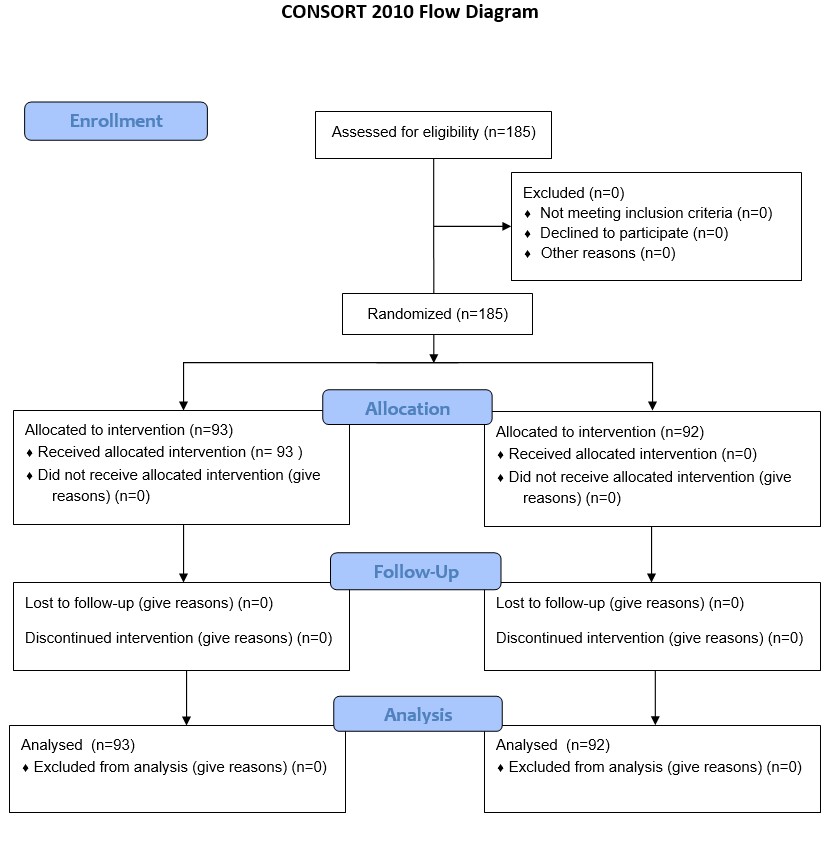

Supplement: Supplementary file 1 [file Data_Sheet_1.zip › Figure 8.jpg]
